# Bornean orangutan nest identification using computer vision and deep learning models to improve conservation strategies

**DOI:** 10.7717/peerj.20333

**Published:** 2025-12-03

**Authors:** Donna Simon, Keeyen Pang, Rayner Bili, Song-Quan Ong, Henry Bernard

**Affiliations:** 1 WWF-Malaysia, Kota Kinabalu, Sabah, Malaysia; 2 Institute for Tropical Biology and Conservation, Universiti Malaysia Sabah, Kota Kinabalu, Sabah, Malaysia; 3 Intrajasa Sdn.Bhd, Sandakan, Sabah, Malaysia; 4 Sabah Forestry Department, Sandakan, Sabah, Malaysia

**Keywords:** *Pongo pygmaeus*, Artificial intelligence, Population survey, Drone, Ecology

## Abstract

**Background:**

Regular population surveys are crucial for the evaluation of conservation measures and the management of critically endangered species such as the Bornean orangutans. Uncrewed aerial vehicles (UAV) are useful for monitoring orangutans by capturing images of the canopy, including nests, to monitor their population. However, manually detecting and counting nests from UAV imagery is time-consuming and requires trained experts. Computer vision and deep learning (DL) models for image classification offer an excellent alternative for orangutan nest identification.

**Methods:**

This study investigated DL for nest recognition from UAV imagery. A binary dataset (“with nest” and “without nest”) was created from UAV imagery from Sabah, Malaysian Borneo. The images were captured using a fixed-wing UAV with a complementary metal-oxide semiconductor camera. After image augmentation, 1,624 images were used for the dataset and further split into 70% training, 15% testing and 15% validation for model performance evaluation, *i.e.*, accuracy, precision, recall and F1-score. Four DL models (InceptionV3, MobileNetV2, VGG19 and Xception) were trained to learn from the labeled dataset and predict the presence of nests in new images.

**Results:**

The results show that out of four DL models, Inception V3 has the best model performance with more than 99% accuracy and precision, while VGG19 has the lowest performance. In addition, gradient-weighted class activation maps were used to interpret the results, allowing visualization of the regions used by InceptionV3 and VGG19 for classification. This study shows the potential of integrating DL into orangutan conservation, particularly in monitoring the orangutan population in the protected environment. Future research should focus on the automatic detection of nests to improve UAV-based monitoring of orangutans.

## Introduction

All three orangutan species (Sumatran orangutan - *Pongo abelii*, Bornean orangutan - *Pongo pygmaeus* and Tapanuli orangutan - *Pongo Tapanuliensis*), which occur on Borneo and Sumatra, have been listed as “Critically Endangered” on the International Union for Conservation of Nature (IUCN) Red List since 2016, as their populations have declined sharply (Ancrenaz et al., 2023). These population declines are primarily due to habitat loss, degradation and fragmentation, as well as retaliatory killings due to conflict with humans (Ancrenaz et al., 2023). In Sabah, in Malaysian Borneo, several measures have been taken to protect orangutans, including the restoration of forests in degraded areas such as the Bukit Piton Forest Reserve (Mansourian, Vallauri & France, 2020), the expansion of fully protected areas to 30% and a commitment to sustainable timber production (Simon, Davies & Ancrenaz, 2019). In addition, the 10-Year Action Plan for Sabah’s Orangutans (2020–2029) has been developed to ensure the long-term survival of the species in the region (Sabah Wildlife Department, 2020). Continuous monitoring is crucial to evaluate population trends and assess the effectiveness of these conservation measures (Piel et al., 2022).

Orangutans are primarily found in lowland tropical rainforests (<1,000 m altitude), where they spend most of their time in the forest canopy (Manduell, Harrison & Thorpe, 2012). They construct new nests each day, with juveniles relying on their mothers to build them (Permana et al., 2024). These nests are used for both night-time sleeping and daytime resting (Casteren et al., 2012). Since observing orangutans directly is difficult due to the dense canopy and their elusive nature, researchers often monitor populations by counting nests, which serve as reliable indicators of their presence (Kühl et al., 2008; Santika et al., 2019). Population estimates are derived from nest densities (nests per km^2^), which are converted into orangutan numbers using established statistical methods (Ancrenaz et al., 2005; Kühl et al., 2008; Pandong et al., 2018).

Orangutan nests are distinct from those of other animals. Orangutans typically build their nests in the upper canopy, around 11–20 m above the ground (Casteren et al., 2012), and the nests are about 100 cm wide to accommodate their large body size (Kamaruszaman et al., 2018). The nest’s base is made from thick branches, with thinner branches twisted and bent but not fully broken. This partial break, known as a “greenstick fracture,” is unique to orangutan nests (Casteren et al., 2012). Leaves are added to form a flat sleeping platform. Orangutan nests are usually oval and asymmetrical, with the long axis oriented towards the tree trunk (Biddle, Deeming & Goodman, 2014). While most nests are built in the upper canopy, they can also be found at branch ends or close to the main tree stem (Rayadin & Saitoh, 2009).

Orangutan population and density is usually estimated using two indirect nest counting methods: Standing Crop Nest Count (SCNC) and Marked Nest Count (MNC) (Spehar et al., 2010). SCNC involves conducting a single survey along transects to count visible nests and estimate orangutan density using the formula *D* = *d*/(*p* × *r* × *t*), where *D* is the orangutan density, *d* is the nest density, *p* is the proportion of nest builders, *r* is the nest production rate and *t* is the nest decay rate (Ancrenaz et al., 2005). While SCNC is efficient and integrates data over time, its accuracy is highly dependent on the estimation of *t*, which can vary greatly and lead to unreliable results if not measured correctly. In contrast, MNC avoids the need for a decay rate by tracking newly built nests between repeated surveys, making it better suited to detecting population trends over time. However, MNC is more labor intensive, especially in terms of resources for counting orangutan nests.

**Figure 1:**
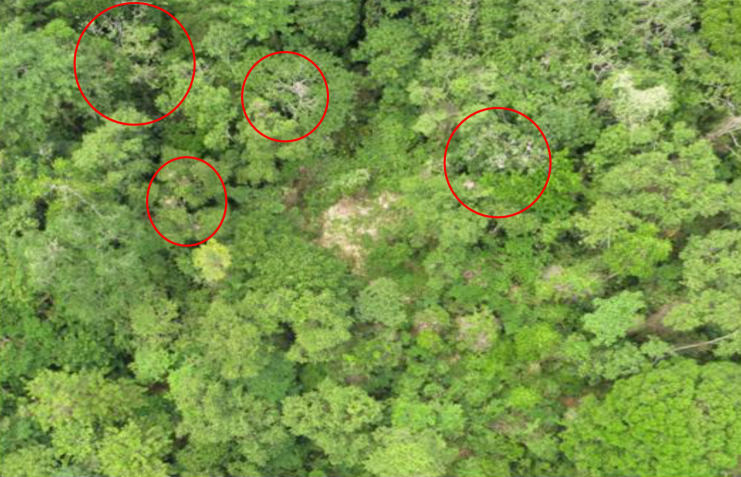
Example images for a drone image with orangutan nests circled in red.

Various methods are used to count orangutan nests, including ground-based nest surveys (Pandong et al., 2018; Santika et al., 2019), helicopter surveys (Ancrenaz et al., 2005; Payne, 1988; Simon, Davies & Ancrenaz, 2019), and the latest technology involving uncrewed aerial vehicles (UAVs) or drones (Hanggito, 2020; Milne et al., 2021; Wich et al., 2015). Among these methods, drones are becoming increasingly important as they are relatively inexpensive compared to helicopters and can capture images or time-lapse video from the forest canopy, allowing many hard-to-access areas to be studied (Wich & Koh, 2018). In contrast to ground and helicopter surveys, where nests are detected through direct field observations, drone imagery requires careful examination of each image on a computer to identify nests. As nests decay, the fresh green foliage withers and turns brown, making them stand out more clearly against the surrounding green canopy in the images (Fig. 1). During manual nest identification, each nest is marked or labelled and then counted across all images. This allows researchers to calculate nest density, which can be used to estimate the orangutan population size.

To classify the images, it is important to consider the canopy classification perspective. Although nests made of branches and leaves can be distinguished from healthy trees as they decay over time (Casteren et al., 2012), a key challenge in using drone imagery to explore orangutan nests is that labeling nests from large volumes of image data still relies heavily on human experts, making the process tedious and time-consuming (Milne et al., 2021; Wich et al., 2015). Therefore, there is a need for an alternative method to identify nests from drone imagery that is as effective as, if not more effective than, human expertise for nest detection.

The integration of artificial intelligence (AI), in particular machine learning (ML), offers an alternative approach to improve the efficiency of orangutan nest detection in drone imagery. This study focuses on supervised learning, where algorithms are trained on labeled/annotated datasets to identify nests based on features such as color, texture, and shape in aerial imagery. Labeled data is essential for developing accurate models. Although ecological studies and ML models use similar evaluation measures such as accuracy and precision, their definitions differ: Ecological accuracy refers to the estimation of population values, while ML accuracy and precision are derived from confusion matrices reflecting true and false classifications (Lebovitz, Levina & Lifshitz-Assaf, 2021). Deep learning (DL), a subset of ML, has been demonstrated as a model for image classification (Pearse et al., 2021). Applications in ecology include identifying tree species or understanding orangutan nesting behavior by combining remote sensing data such as Light Detection and Ranging (LiDAR) with behavioral patterns (Davies et al., 2019).

DL models are well suited for image classification as the architecture uses multiple layers of neural networks consisting of perceptions to model complex data (*e.g.*, images with different colour channels) by learning features from images and making predictions (Smith et al., 2018). Further details on how the DL model works can be found in Chatzilygeroudis, Hatzilygeroudis & Perikos (2021), Purwono et al. (2022), the protocol article by Isawasan et al. (2023) and Madhavan & Jones (2024). In image processing, DL is widely used for image classification and object detection in ecological studies, such as species identification, animal behavior classification and species diversity estimation from camera traps, video and audio recordings (Christin, Hervet & Lecomte, 2019). For orangutan studies, Guo et al. (2020) developed Tri-AI, an automatic recognition system that identifies 41 primates and four carnivores with 94% accuracy. In addition, Desai et al. (2023) developed an annotated database of apes in different poses which enables object recognition for behavioral studies of apes in zoos.

Studies on orangutan recognition through computational methods to detect and count orangutan nests remain limited. Nest building, a unique daily behavior of orangutans for sleeping, offers valuable data for ecological monitoring, and by integrating DL techniques, it could enhance population monitoring efforts. Amran et al. (2023) initiated the study on the use of ML—support vector machine (SVM)—in classifying the objects on the aerial images into branches, buildings and orangutan nests; Teguh et al. (2024) provided the most recent study (at the time of writing this manuscript) on orangutan detection using DL model, the You Only Look Once (YOLO) version 5 with 414 labelled orangutan nests and achieved a precision of 0.973 and a recall of 0.949. However, Teguh et al. (2024) applied an object detection algorithm and demonstrated the effectiveness of a DL model, but this raises additional questions. For instance, YOLO typically identifies and classifies objects in a single step, but alternative classification algorithms may offer improved performance. As biologists and ecologists, it is crucial not to treat these tools as black boxes. This study focuses on interpreting the outputs to gain insight into how DL models ‘visualize’ image patterns and identify the features utilized by neural network layers to classify tree canopy patterns as ‘with nest’ or ‘without nest.’ Understanding this process is essential for accurate ecological interpretation.

Therefore, this study aims to evaluate the effectiveness of different DL models in detecting orangutan nests from aerial images captured at two orangutan sites (Sepilok Virgin Jungle Reserve and Bukit Piton Forest Reserve) in Sabah, Malaysia. More importantly, this study visualizes the model layers to understand how the features and characteristics of orangutan nests are ‘learned’ by the models. Specifically, the aim of this study is to create a labelled dataset of drone images containing both the presence and absence of orangutan nests, and subsequently to develop and compare four DL models for detecting and predicting nest presence from drone images. Additionally, gradient-weighted class activation maps (Grad-CAM) are presented to visualize the activation region used by the models to distinguish orangutan nests from the tree canopy.

## Materials & Methods

### Study site

**Figure 2:**
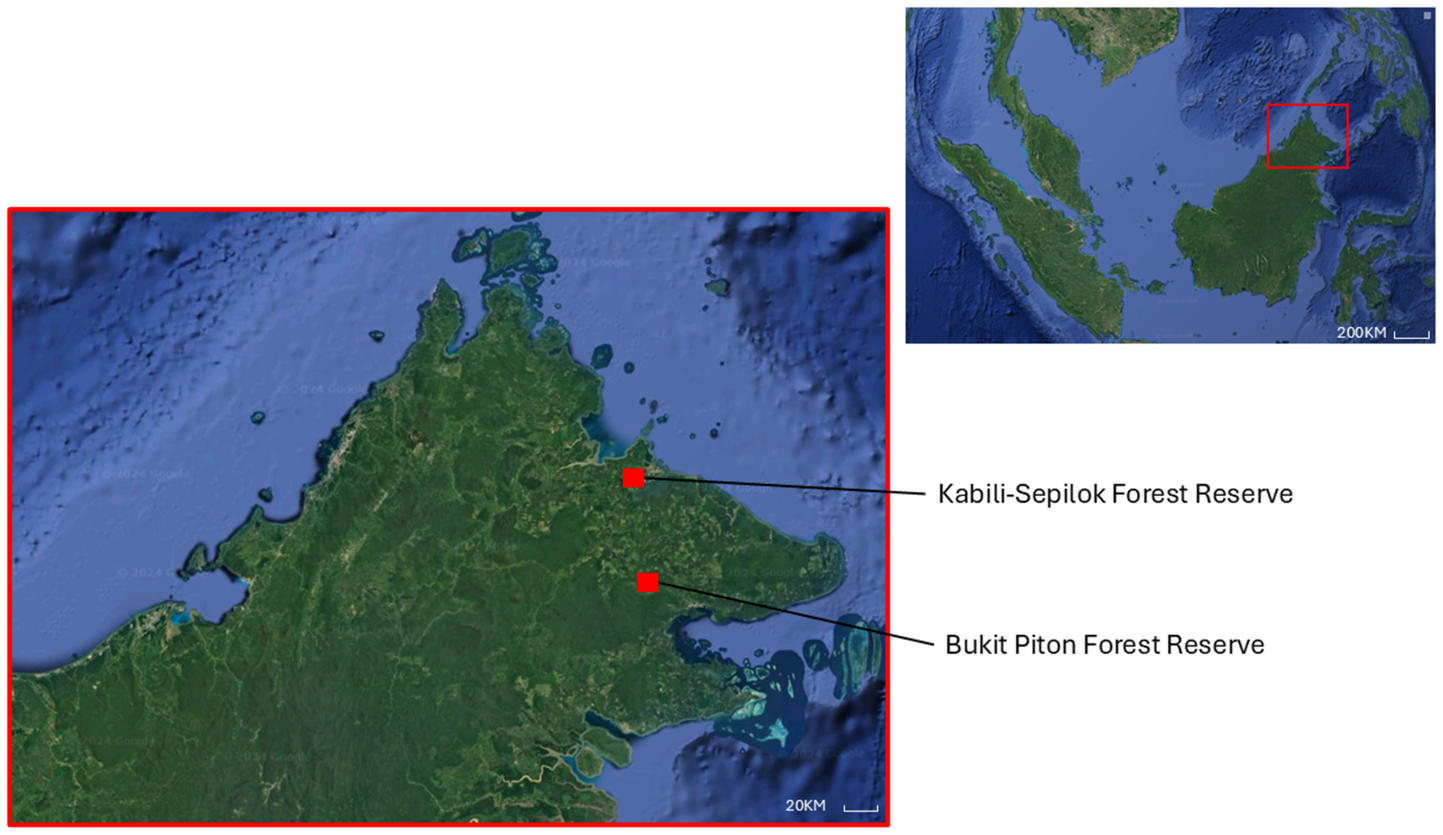
Location of Sepilok Virgin Jungle Reserve and Bukit Piton Forest Reserve in the Malaysian state of Sabah, Northern Borneo.

Drone surveys were conducted in Sepilok Virgin Jungle Reserve (VJR) (5.865092291902751, 117.94834681807893) and Bukit Piton Forest Reserve (FR) (5.110623189107781, 118.00774396297084) in Sabah, Malaysia (Fig. 2). Both reserves are under the management of the Sabah Forestry Department and are known habitats for orangutans. It is estimated that there are about 200 (Range: 100–300) orangutans in Sepilok (Ancrenaz et al., 2005) and 176 (Range: 119–261) orangutans in Bukit Piton (Simon, Davies & Ancrenaz, 2019). The Sepilok VJR covers an area of approximately 40 km^2^ and is characterized by lowland dipterocarp and heath forests (Ball et al., 2023). The reserve has been designated as a protected area where logging is strictly prohibited to keep the forest canopy intact. In contrast, Bukit Piton FR, which consists mainly of dipterocarp lowland rainforest and is about 120 km^2^ in size, is severely degraded due to heavy logging and forest fires in the past. In 2008, a large-scale project was initiated to restore the forest for orangutans, and the area was declared a protected forest in 2012. Since then, the forest has slowly regenerated, with fast-growing tree species being used by the orangutans for nesting just three years after planting (Mansourian, Vallauri & France, 2020).

### Study duration

The Sepilok VJR survey was conducted in July 2015 and covered an area of approximately 0.5 km^2^. A total of three flight missions were conducted to complete the survey yielding 1,720 images. The Bukit Piton FR survey was conducted in January 2016 and covered an area of approximately 0.5 km^2^ resulting in 1,911 images. A total of four missions were flown to survey the area in Bukit Piton FR. All surveys were conducted in the morning on a sunny day (temperature 26−33 °C, relative humidity 73–80%).

### Equipment

This study uses UAV imagery captured by a fixed-wing drone and compiled by ConservationDrones.org (https://conservationdrones.org/), which focuses on the use of low-cost drones for conservation applications by conservationists and researchers worldwide. The fixed-wing drone was equipped with an FX-71 body housing a Canon PowerShot S100 digital camera with RBG CMOS sensor (12.1 megapixels, 1/1.7- inch CMOS sensor, a 24–120 mm f/2.0–5.9 lens and the DIGIC 5 image processor, Canon Malaysia). The drone was flown with a flight task that was at least 100 m away from the highest point, which was determined using the Digital Elevation Model (DEM). The waypoint map was created using WaypointMap software (https://www.waypointmap.com/), with 80% of the flight path overlapping with the vertical and horizontal image and time-lapse images captured at 3-second intervals. The aim of this study is to compare DL classifier models used to solve a classification problem where the whole image serves as the target object (nest or non-nest). This is in contrast to another type of DL model, object detection models, where the algorithms usually have to localize and classify an image (two tasks and therefore more computationally complex). Therefore, the dataset was created by combining images from both sites and annotated by six human experts from WWF, who have at least two years of experience in studying nests, and categorized into two binary classes, *i.e.,* images with nests and images without nests. This binary classification is needed to train the model and determine whether an image contains an orangutan nest or not. The field study and the use of the drone for aerial images were conducted in 2014 with the permission of the Sabah Forestry Department under reference number (JPHTN/PP 100 − 22/4/*K* LT.11(44)).

### Pre-processing of the data and categorization

The images of Sepilok VJR (1,720) and Bukit Piton FR (1,911) were mostly repeated and overlaid to create a combined map for the geospatial study. For this study, only one image was selected from the repeated images of the time-lapse photography. In addition, the entire images were classified as either “with nest” or “without nest” using the image classification task. For images with multiple nests, the images were therefore pre-processed by cropping out the nest and labeling it as “with nest”. The total number of drone images from Sepilok and Bukit Piton amounted to 406 images, which were divided into two classes, *i.e.,* with nest (162 images) and without nest (244 images) (Table 1). The original image resolution was 4,000 × 3,000 px. at 180 dpi and was normalized to 300 × 300 px for the development of the deep learning model.

**Table 1:** Number of aerial images used in the dataset according to classes and after augmentation.

Classes	Number of images		Train (70%)	Test (15%)	Predict (15%)
	Original	Augmented	Original (Augmented)		
With nest	162	648	113 (452)	25 (100)	24 (96)
Without nest	244	976	171 (684)	37 (148)	36 (144)
Total	406	1,624	284 (1,136)	62 (248)	60 (240)

**Figure 3:**
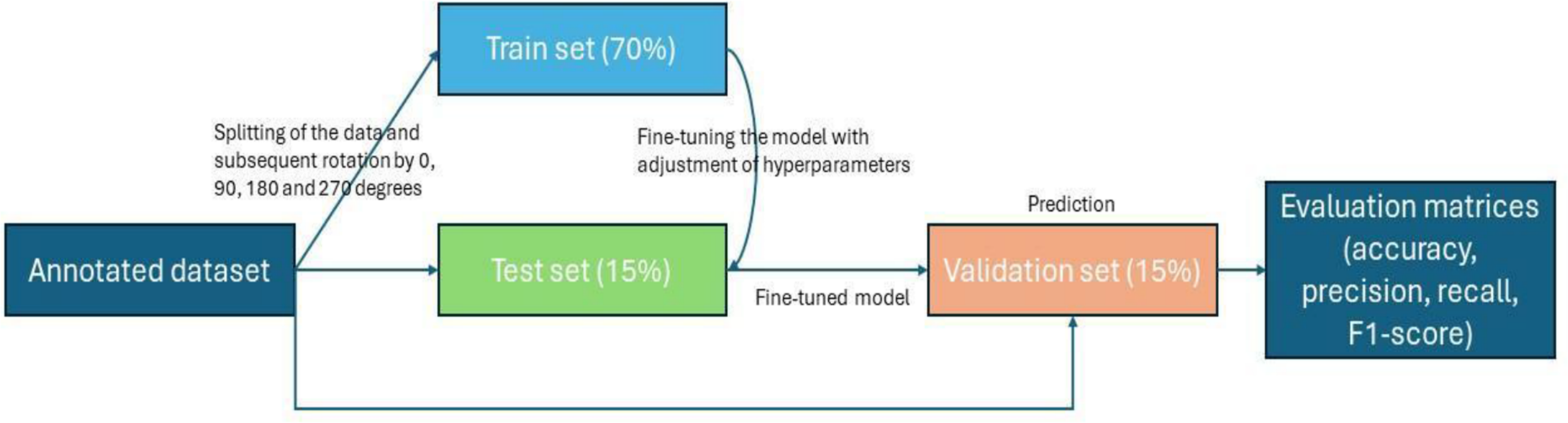
Workflow of image processing, data splitting until performance measure.

Nests from drone images have been identified by six orangutan field specialists, with more than two years of field experience in conducting ground and helicopter nest surveys. The identification of the orangutan nest at the same sites where drone images were captured is also validated with the ground survey data which confirmed the presence of nests through direct observations. Then, the total number of images in each class was divided into three parts, also known as data splitting, with 70% of the total images used for training, 15% for validation and 15% for testing or a 70:15:15 ratio (Fig. 3). The ratio of data splitting is based on the amount of data used for training and evaluation, and reducing the size of the training dataset tends to result in a poorly performing model. Therefore, an international standard of computer vision and DL competition (Fei-Fei, Deng & Li, 2009) was referenced, along with insights from previous studies (Khan & Ullah, 2022; Ong & Hamid, 2022). Data splitting enables the machine to use the training set to obtain the weights and biases for classification. The validation set helped to better generalize the models to new, unseen data and prevent over-fitting while the testing set is to assess the model’s performance. As the number of images was relatively small, each image was subjected to a rotation expansion of 0°, 90°, 180° and 270° and finally the number of images was increased by a factor of four (Ong et al., 2022; Chen et al., 2021), totaling to 1,624 images used for the model development.

### Models development

#### Model build-up

To develop the DL models, the convolutional blocks of the pre-trained convolutional neural networks (CNNs) were unfrozen for retraining purposes (a process in which the weights and biases that the model learns from the ImageNet are unlocked for a customized task, *i.e.,* orangutan nest classification). This was done for four DL architectures—InceptionV3, MobileNetV2, VGG19 and Xception—to optimize them for the specific task of identifying nests from aerial images, as described in Ong et al. (2022). The Keras DL framework was executed on the Google Cloud Platform (https://cloud.google.com/) with an NVIDIA Tesla A100 Graphics Processing Unit (GPU), which was used to train and evaluate the models. The models were trained with the Adaptive Moment Estimation (ADAM) optimizer, which improves the stability and efficiency of the training process and enables efficient learning (Okewu, Misra & Lius, 2020). Three learning rates (0.01, 0.001 and 0.0001) with 32 batches were analyzed. The training process was set to 50 epochs, meaning that the model performed 50 complete iterations through the training dataset (Chatzilygeroudis, Hatzilygeroudis & Perikos (2021)). Increasing the number of epochs allows the model to refine its parameters and could improve its performance. After developing the models, the performance of these models was evaluated using the four metrics of accuracy, precision, recall and F1-score (Table 2) (Kumar et al., 2020). In addition, the mean accuracy (number of correct predictions/total number of images) was compared between the models to test the significance of the four DL models. The code used for the model development was publicly available at GitHub with the link https://github.com/songguan26/Bornean-Orangutan-Nest-.

**Table 2:** Calculation of evaluation matrices (Li et al., 2019).

**Evaluation matrix**	**Evaluation focus**	**Formula**
Accuracy	Accuracy is the proportion of all classifications that were correct, whether positive or negative	$ \frac{\mathrm{TP}+\mathrm{TN}}{\mathrm{TP}+\mathrm{TN}+\mathrm{FP}+\mathrm{FN}} $
Precision	Precision is the proportion of all the model’s positive classifications that are actually positive.	$ \frac{\mathrm{TP}}{\mathrm{TP}+\mathrm{FP}} $
Recall	The recall is the proportion of all actual positives that were classified correctly as positives	$ \frac{\mathrm{TP}}{\mathrm{TP}+\mathrm{FN}} $
F1-Score	The harmonic mean between recall and precision values.	$ \frac{2\mathrm{p}\mathrm{x}\mathrm{r}}{\mathrm{p}+\mathrm{r}} $

**Notes.**

TP true positive TN true negative FP false positive FN false negative p precision r recall

#### Activation map to distinguish orangutan nests from aerial images

To gain further insight into how the neural network in the DL models can recognize the orangutan nest, Grad-CAM was used to visualize the area used by the neural network to classify the orangutan nest with a variety of normal tree canopy backgrounds. In general, one layer at a time was retrieved to extract low- and high-level features. The code used for the model development was publicly available at GitHub with the link https://github.com/songguan26/Bornean-Orangutan-Nest-.

## Results

### Model performance

**Figure 4:**
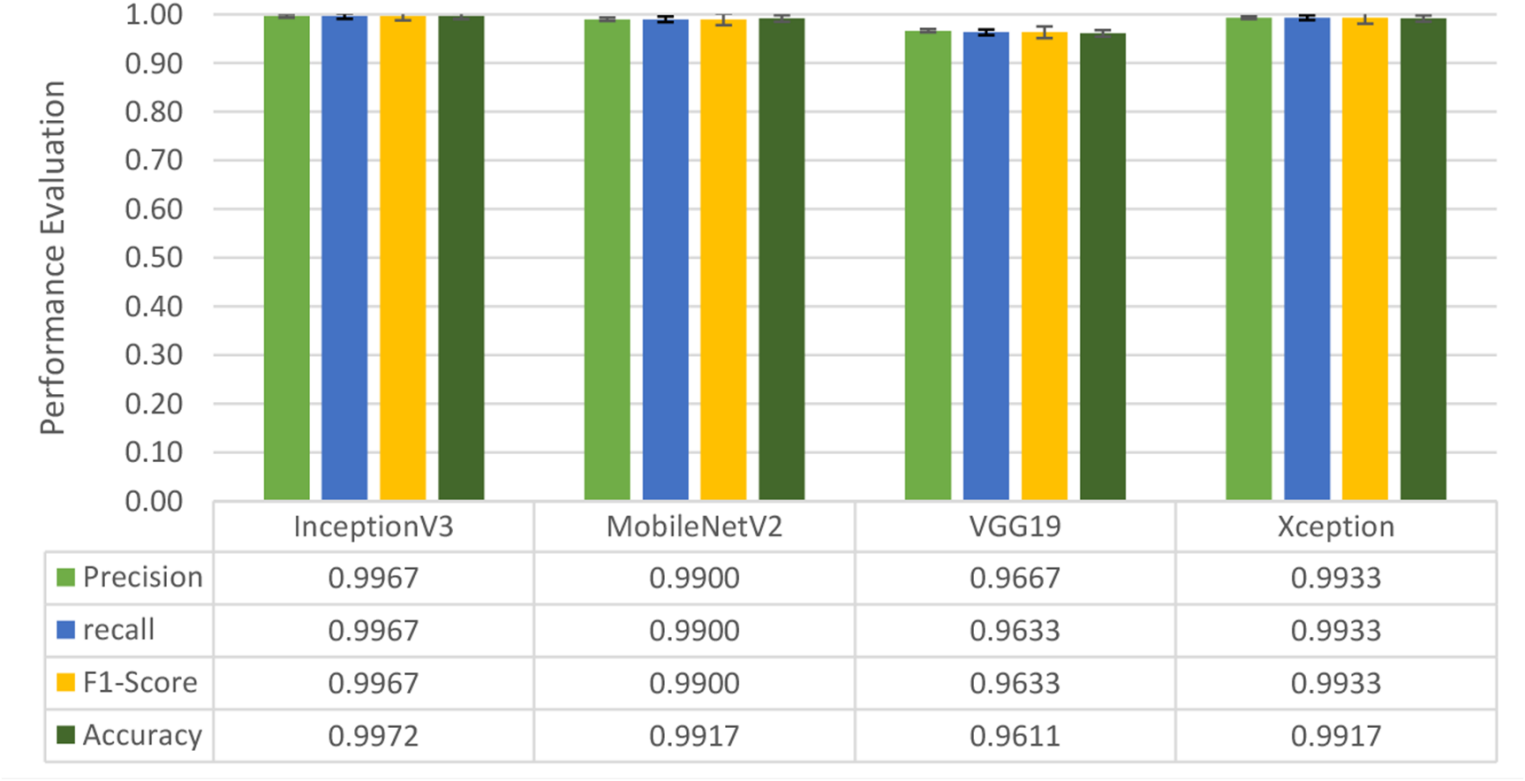
Overall performance of four models. The bar chart represents the mean probability and the error bar represents the standard error (SE).

Four DL models were attempted, and the images were trained, tested and validated for image classification tasks by classifying UAV images into “without nests” and images “with nests” categories. Figure 4, shows the performance of the four models in predicting the images with presence or absence of nests. It can be seen that VGG19 performs lower than the other models. InceptionV3, MobileNetV2 and Xception were ranked first, second and third, respectively. The Shapiro–Wilk normality test was performed to assess the normality of the accuracy values for the models across three learning rates. The results are as follows: InceptionV3 (*W* = 0.75, *p* = 0.0000009), MobileNet (*W* = 0.99, *p* = 0.99), VGG19 (*W* = 0.95, *p* = 0.566) and Xception (*W* = 0.89, *p* = 0.37). Based on these results, only InceptionV3 is not normally distributed (*p* < 0.05). Therefore, a non-parametric test, the Kruskal Wallis H-test, was used to compare the models based on their accuracy values across three LRs. The result of the Kruskal Wallis H-test shows no significant difference (*i.e.,* at the threshold *p*-value <0.05) in the accuracy of the four models at three learning rates (H (3) = 6.751, *p* = 0.087). Additionally, as most of the models are normally distributed except InceptionV3 with a very small *p*-value, the model performance is presented in Fig. 4 using the mean value to better represent the data.

To assess the generalization capabilities of the model—its ability to make accurate predictions on new data (Caro et al., 2022) the training validation accuracy (TVA) and training validation loss (TVL) of the models across three learning rates on the test set were evaluated and presented in Table 2. The new data was validation splits (15%, in section ‘Methodology’) that were never used in the model development. Although the epochs were set at 50, the early-stopping-method was employed—to prevent overfitting and underfitting (Cai et al., 2022) causing the model computation to halt early once the validation accuracy did not improve (epochs indicated in *X*-axis). The results of TVA and TVL (Table 3) show that LR 0.001 generally achieves a balance between efficient training and robust generalization across the models. Whereas, LR 0.01 risks instability and overfitting, which occurs when the model fits the training data too closely and failed to generalize to new data (Charilaou & Battat, 2022). Meanwhile, LR 0.0001 results in slow or failed convergence and underfitting is shown by the poor performance of VGG19 model, which is incapable of learning the patterns in the training data (Jabbar & Khan, 2015).

**Table 3:** Multiple curve diagrams for accuracy and loss for the maximum performance deep learning model on the test set. For training and validation accuracy curve, the *y*-axis is the accuracy in probability and *X*-axis is the epochs. For training and validation loss curve, *y*-axis is the loss in probability, *x*-axis is the epochs.

	**Learning** ** rate**	**Accuracy**	**Loss**
InceptionV3	0.01	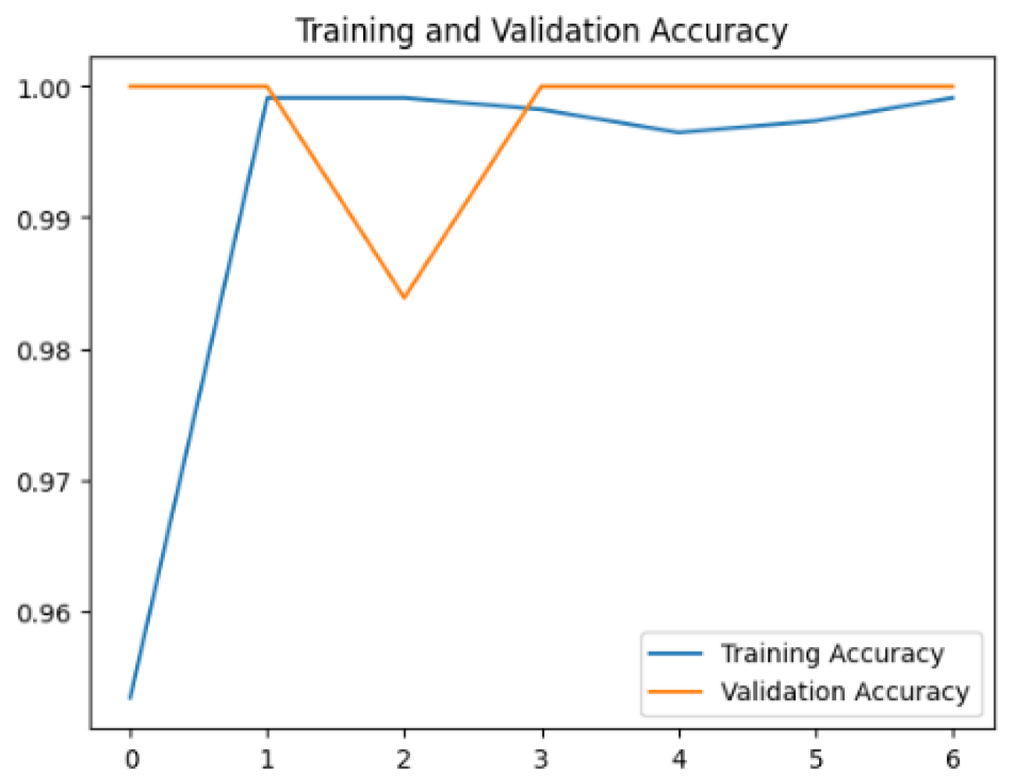	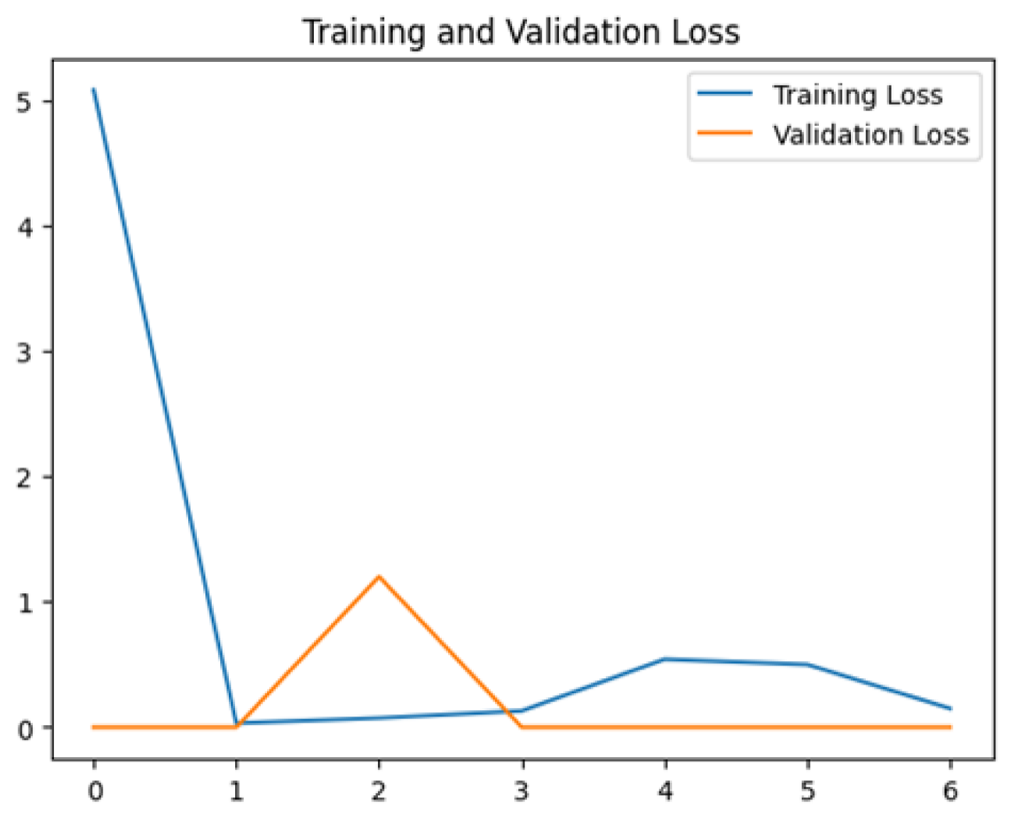
	0.001	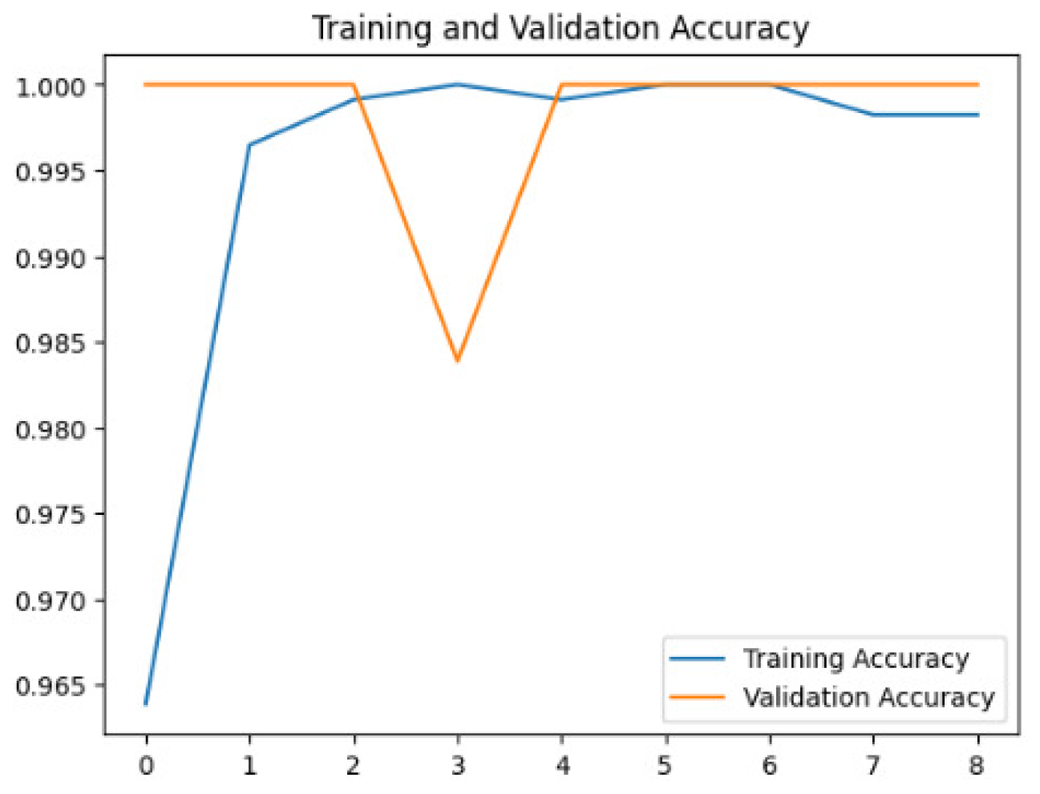	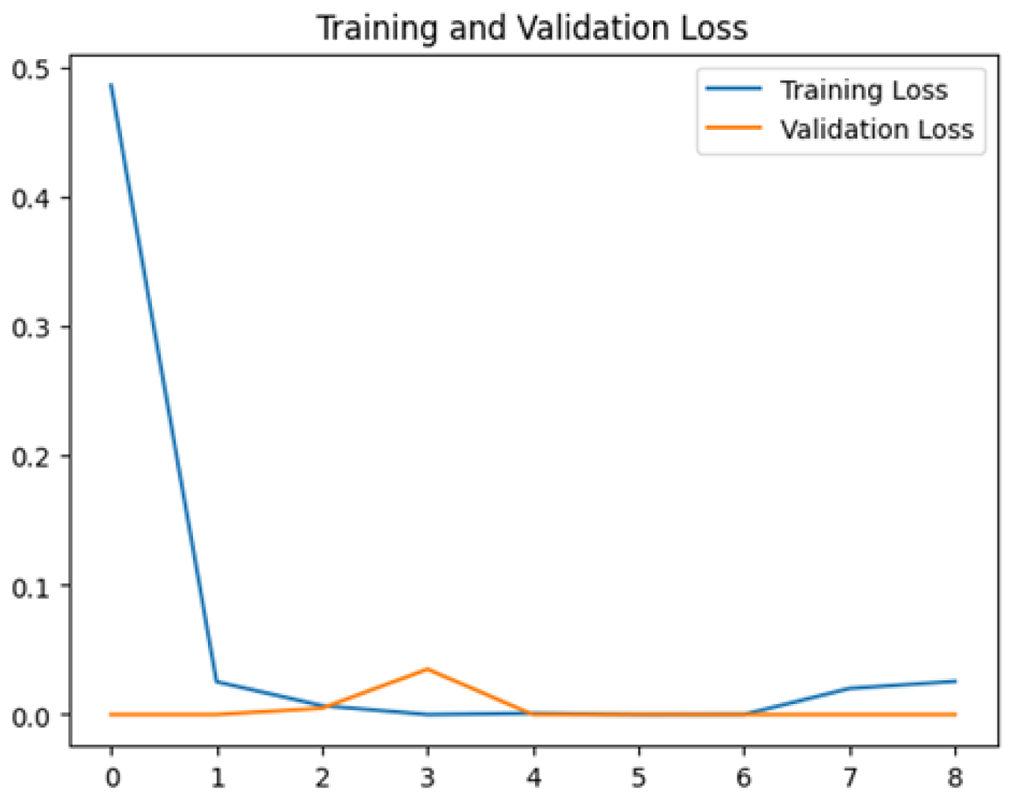
	0.0001	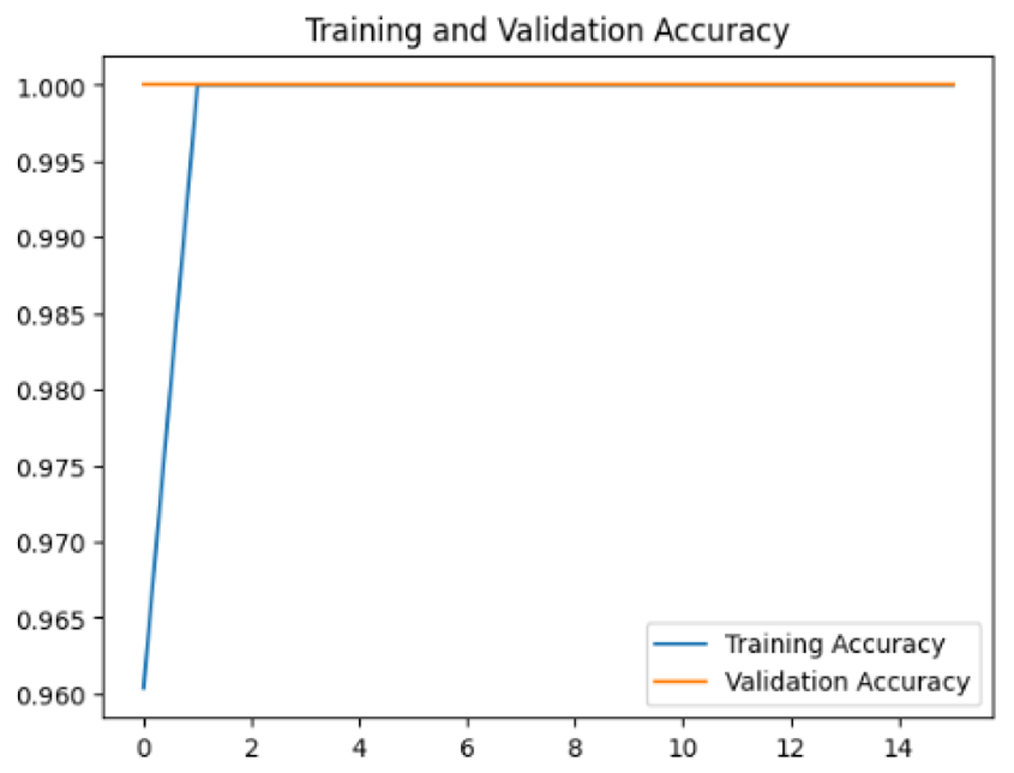	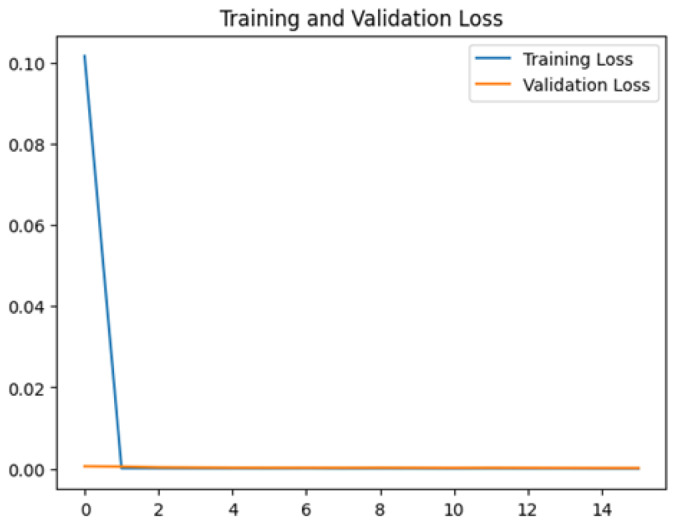
MobileNetV2	0.01	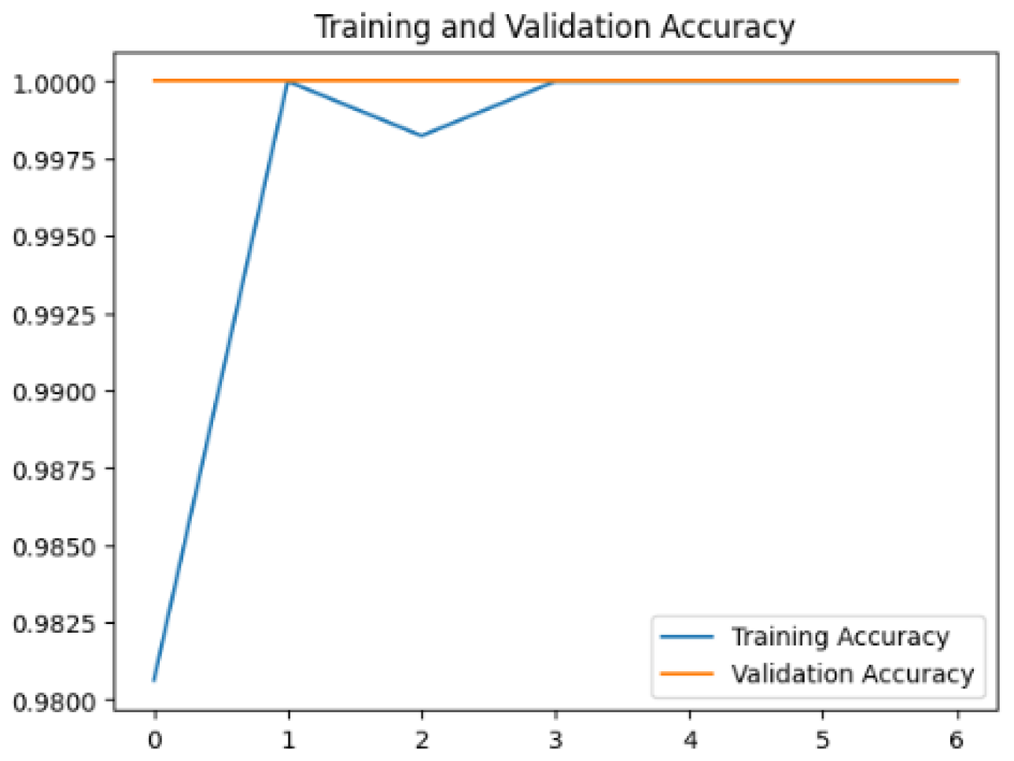	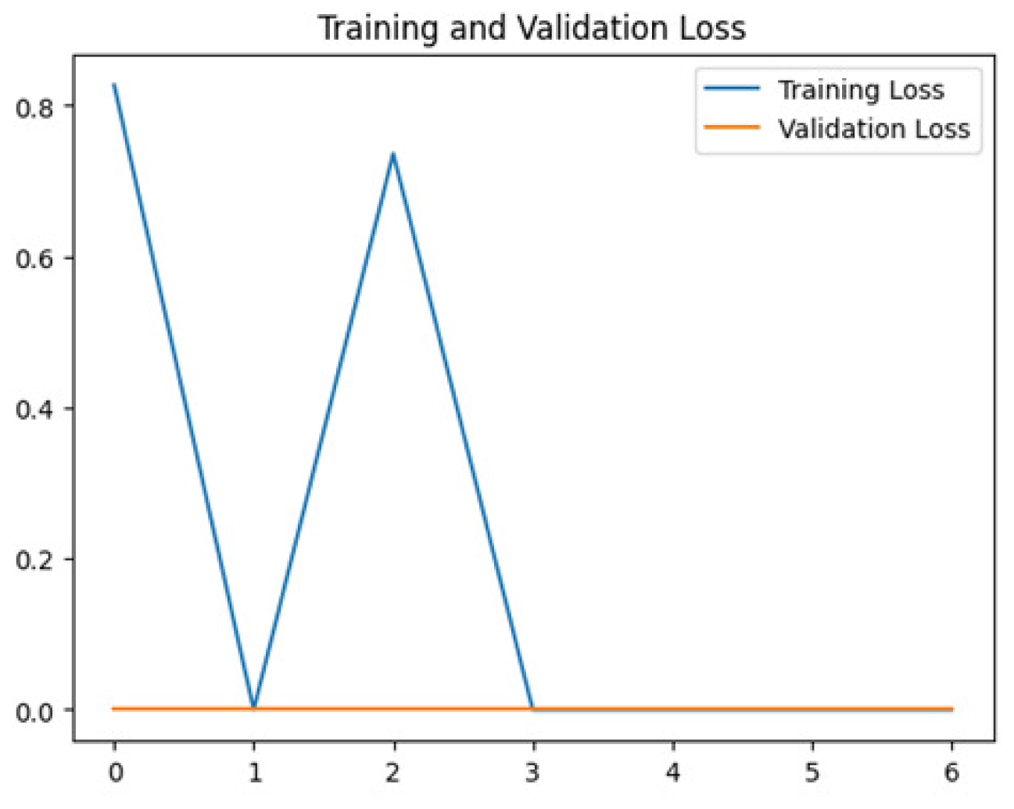
	0.001	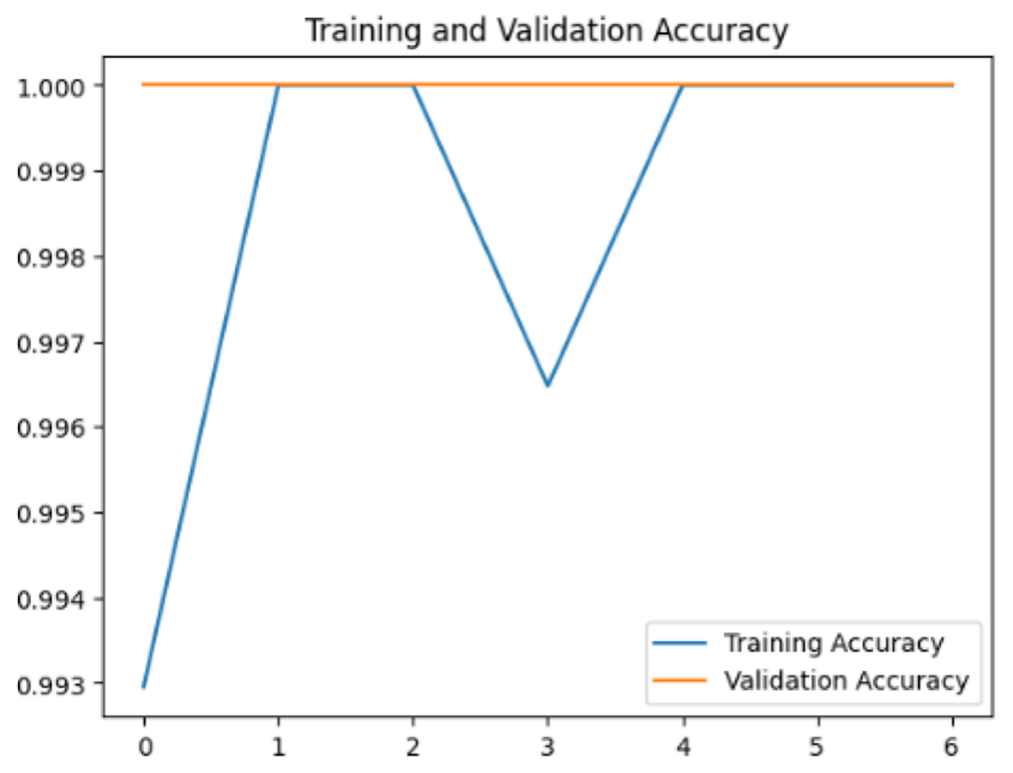	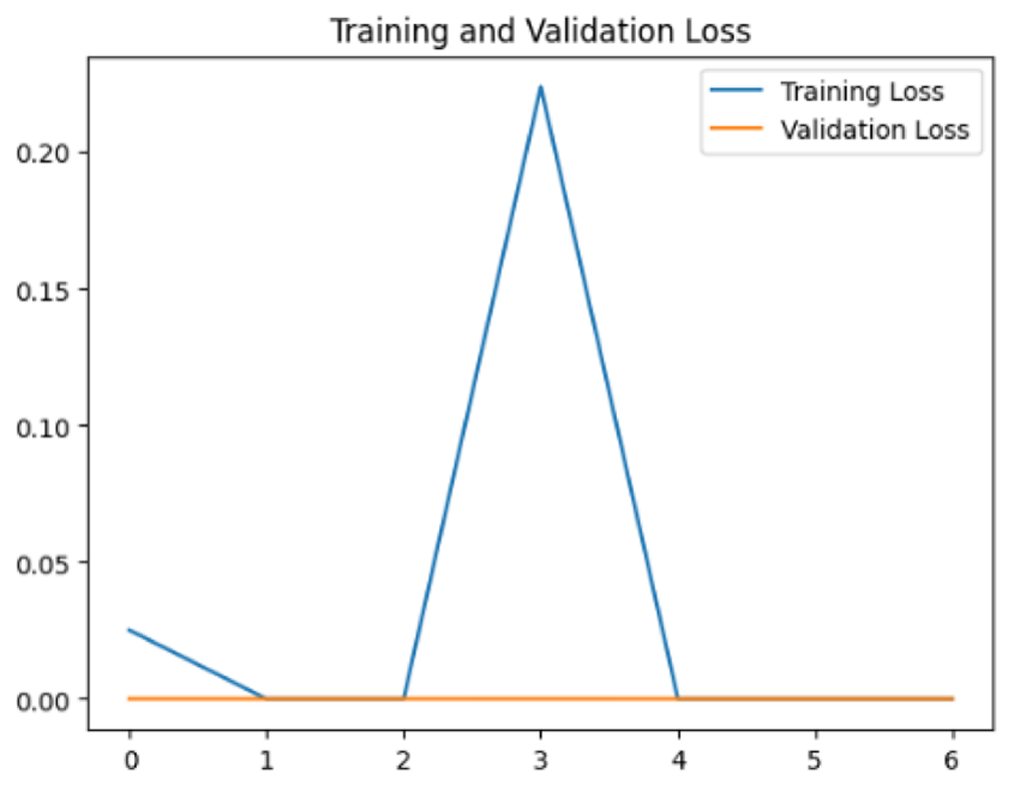
	0.0001	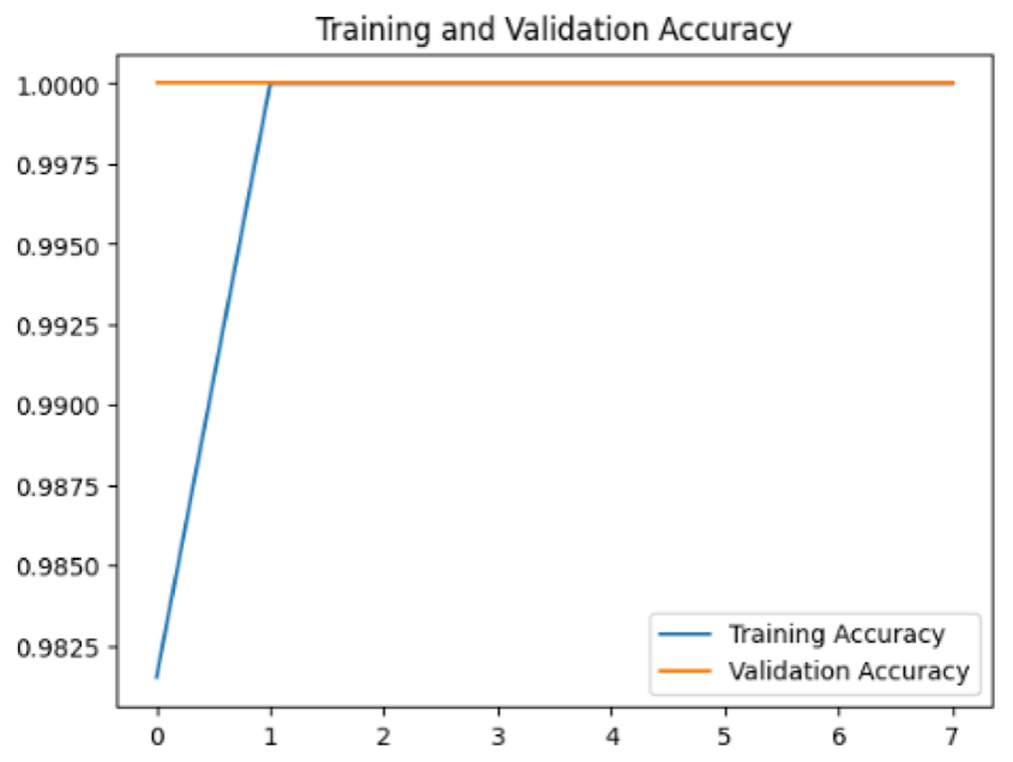	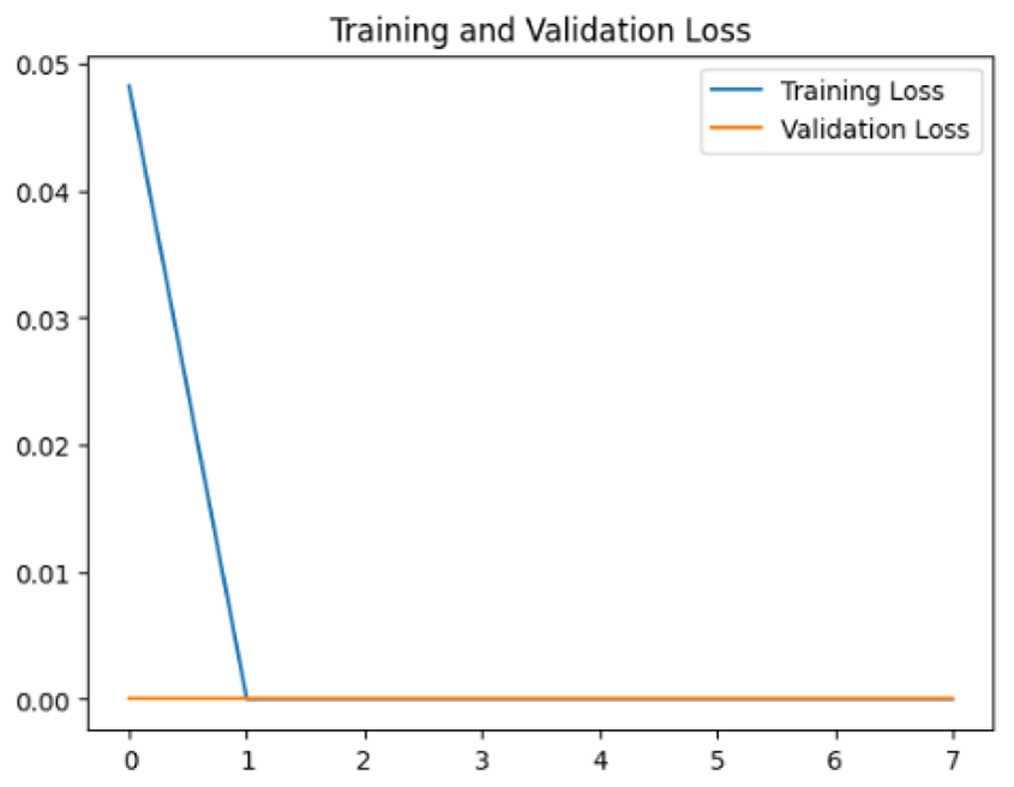
VGG19	0.01	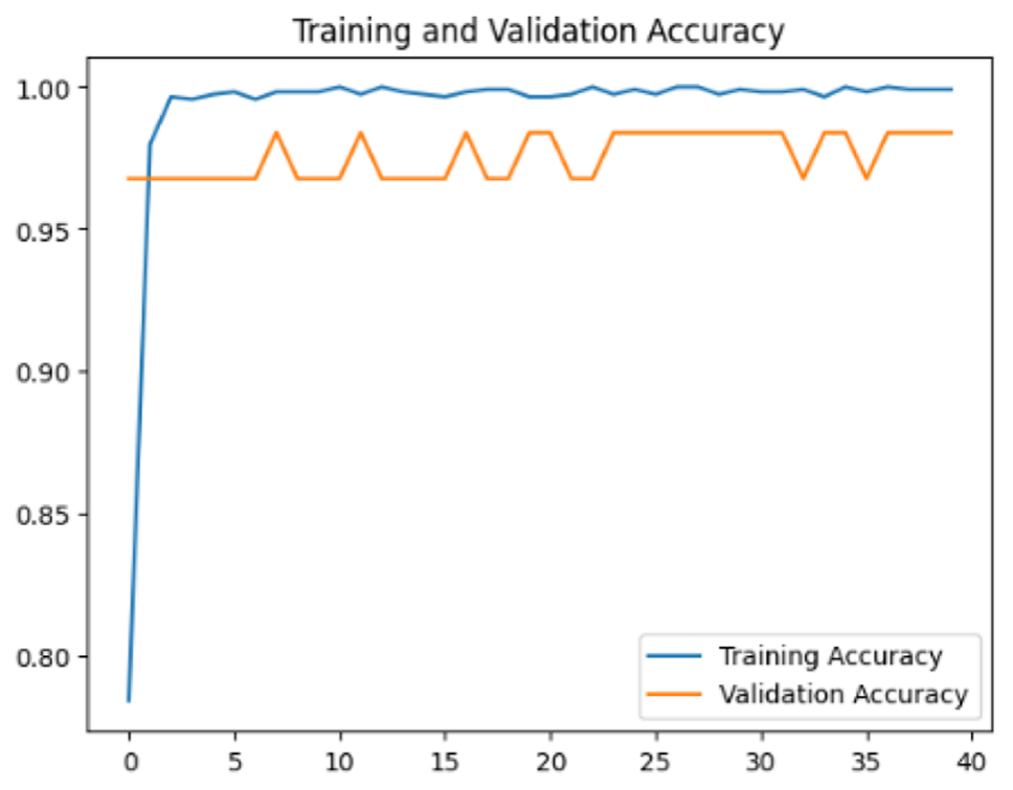	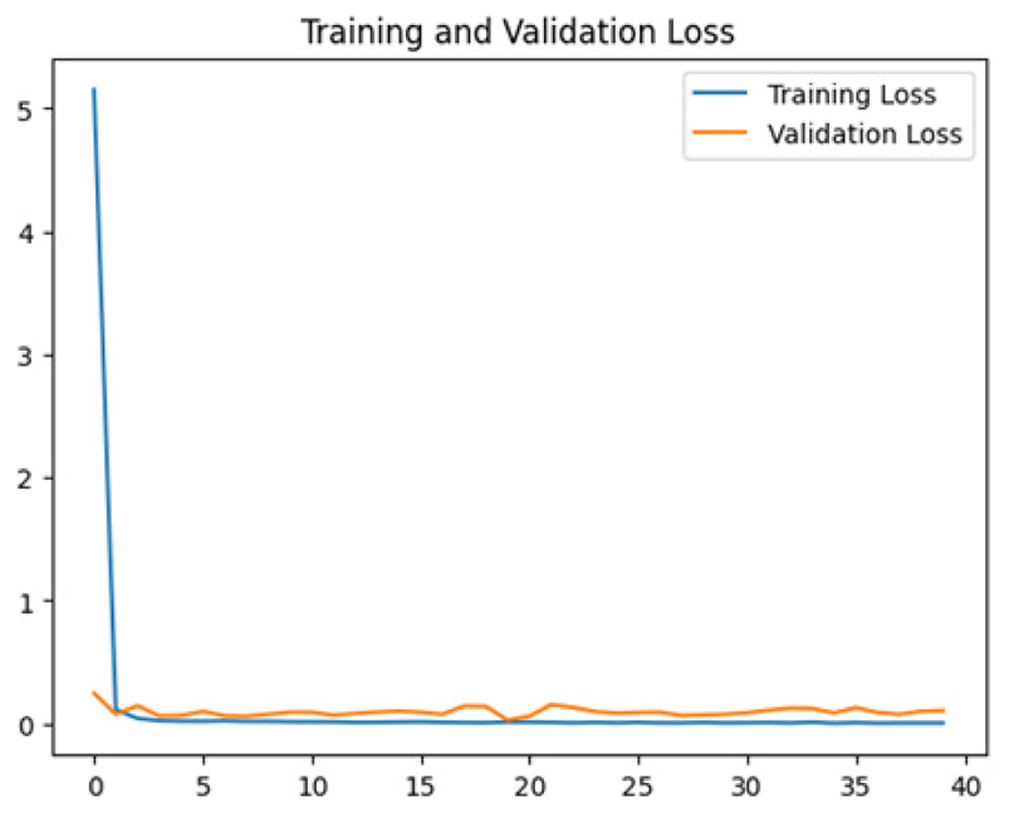
	0.001	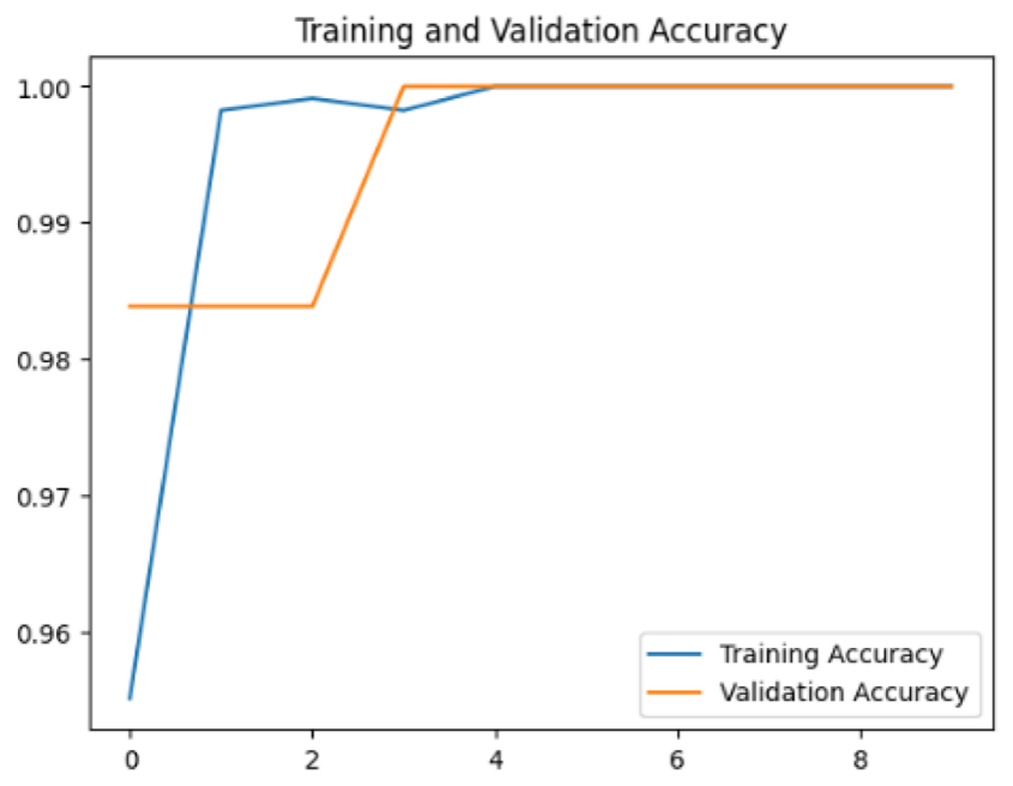	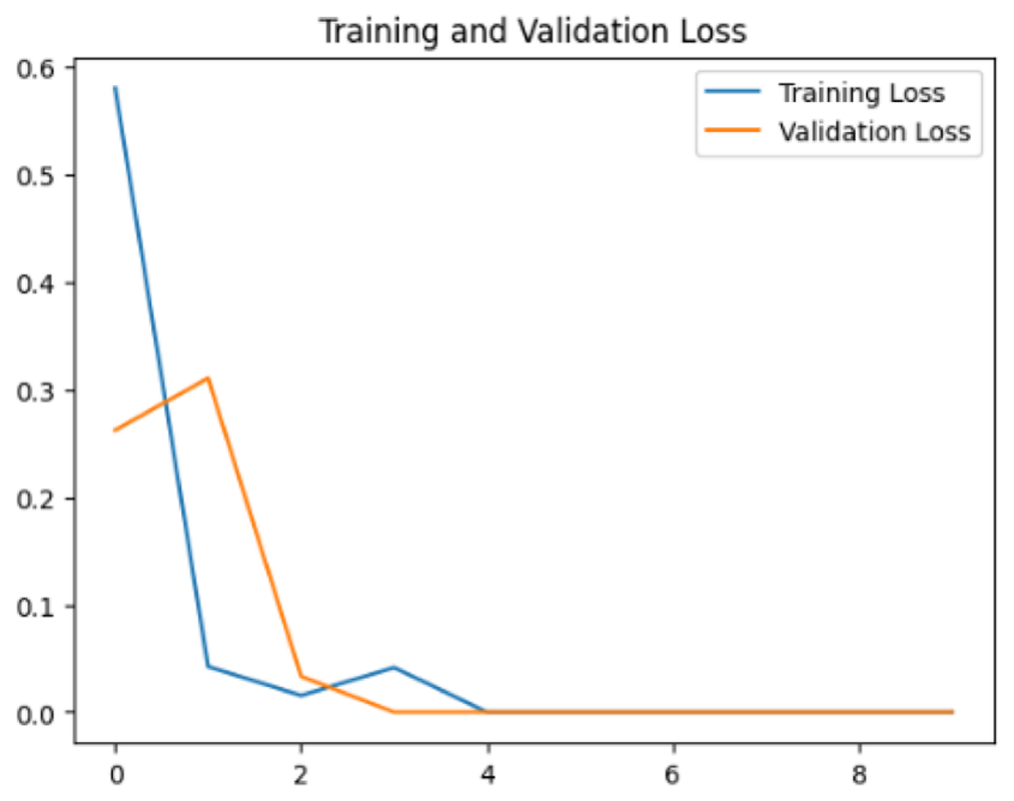
	0.0001	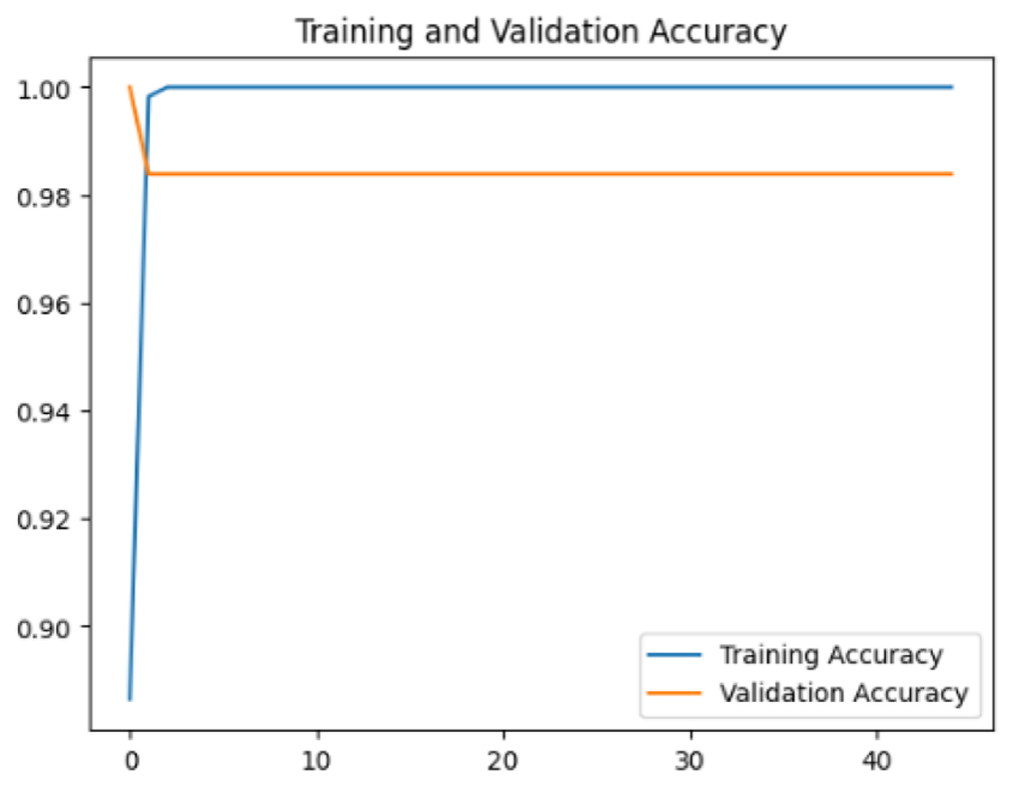	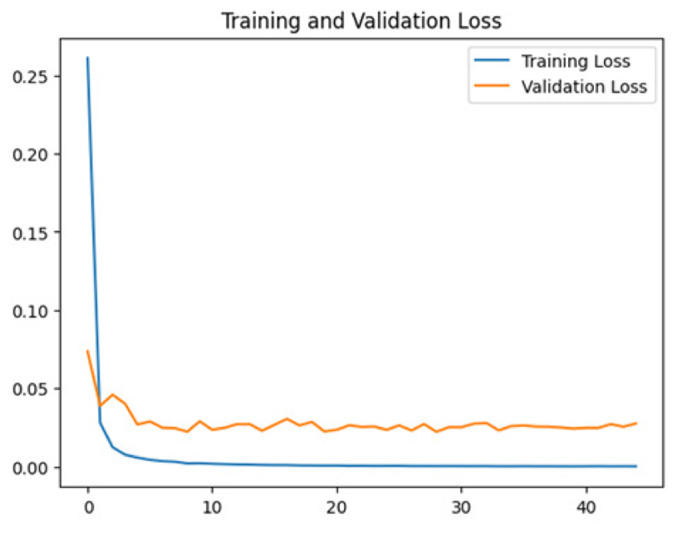
Xception	0.01	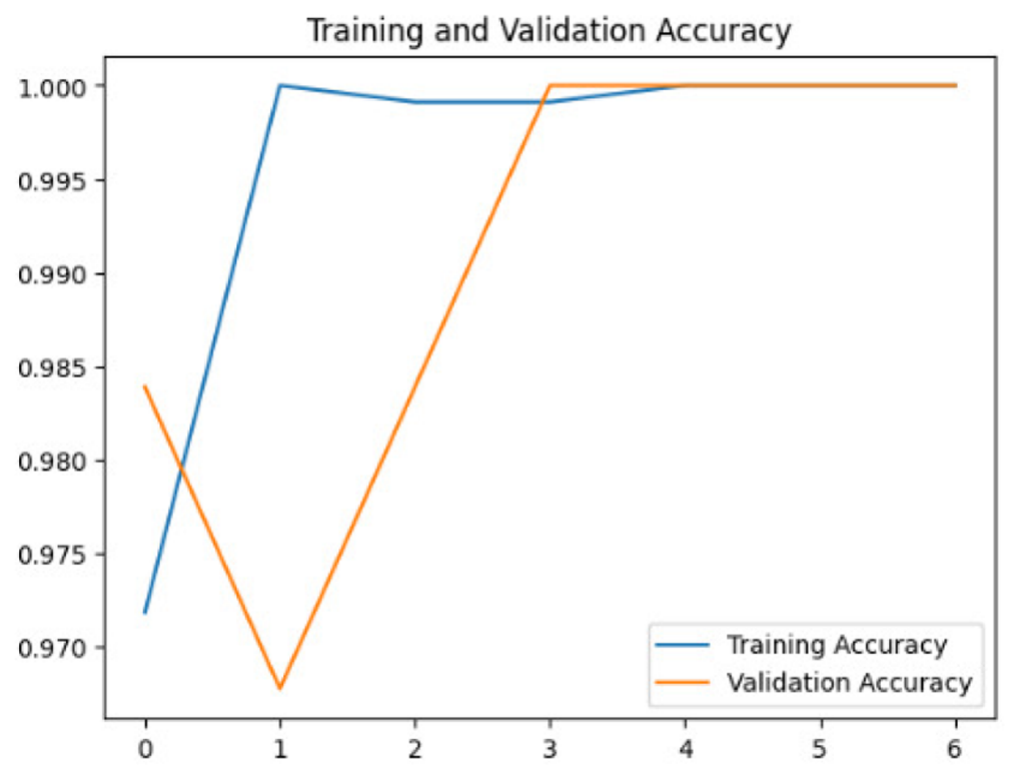	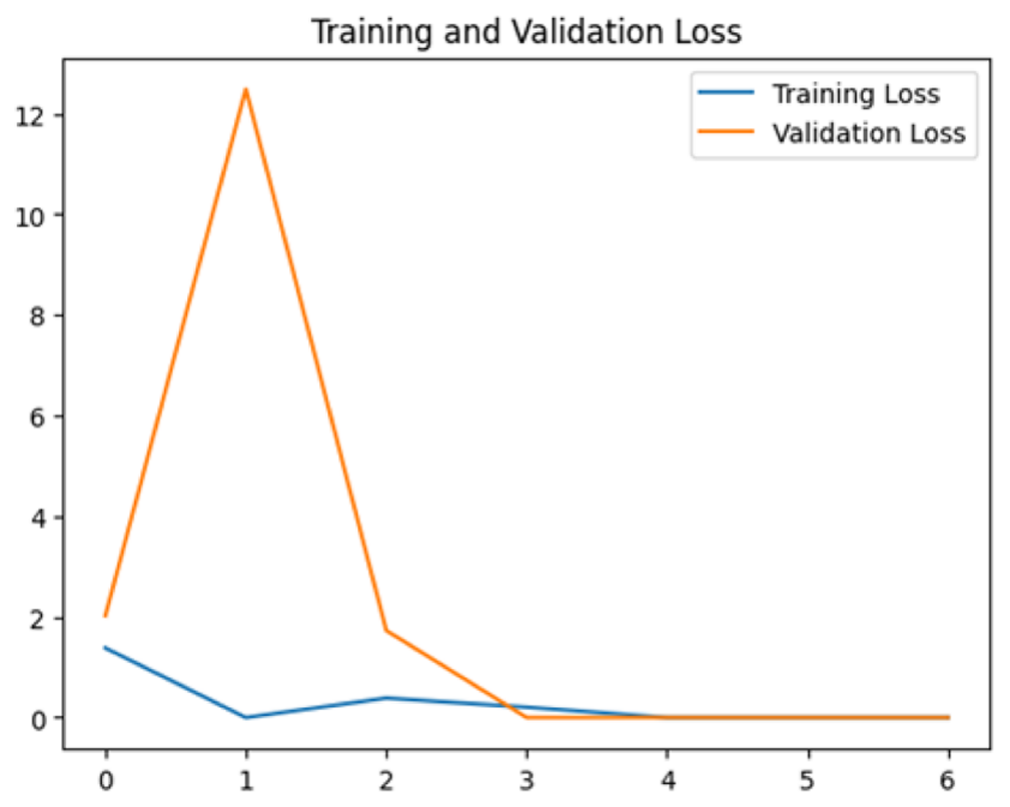
	0.001	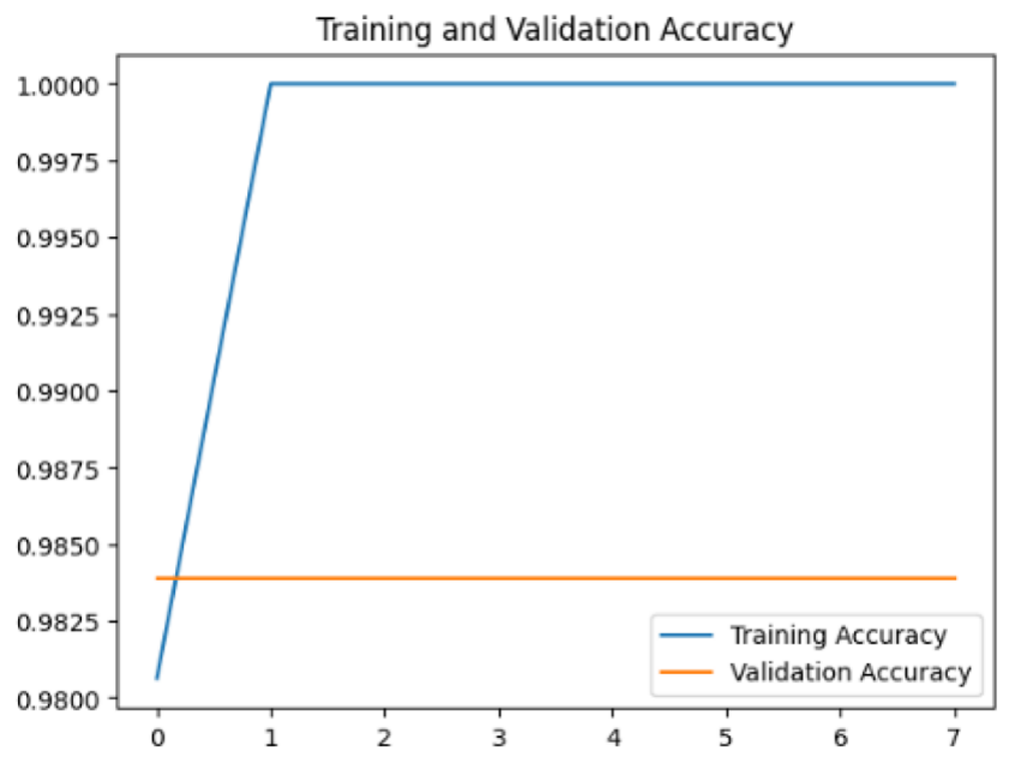	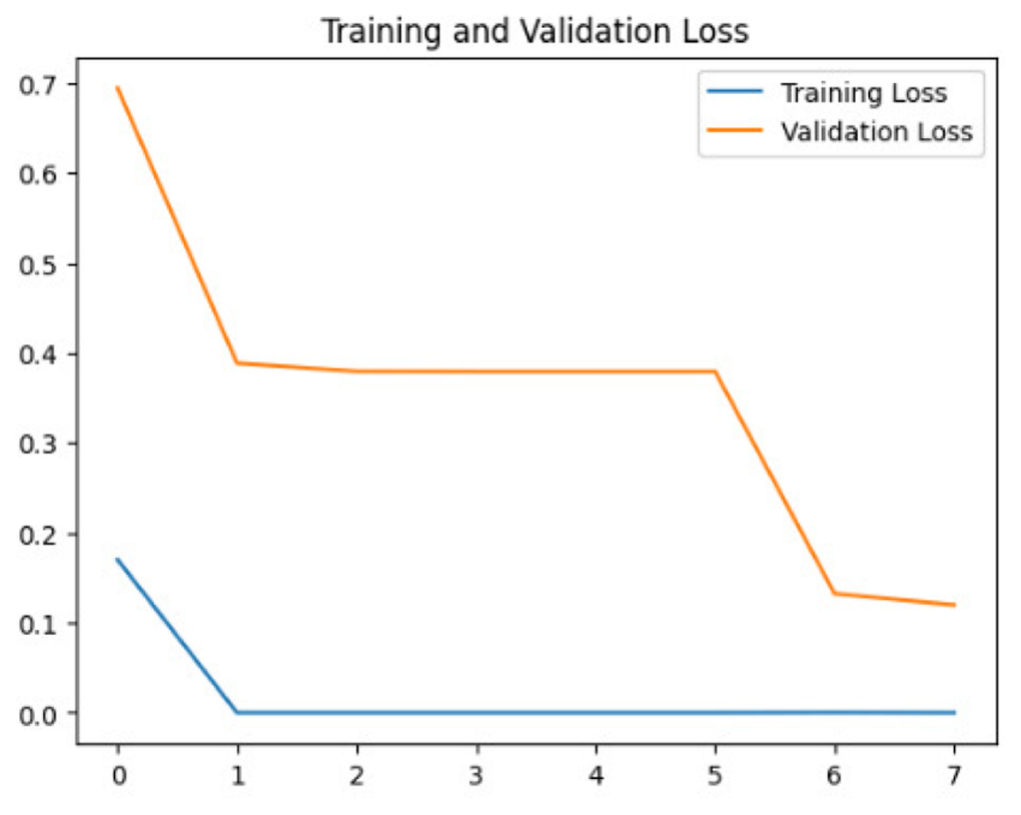
	0.0001	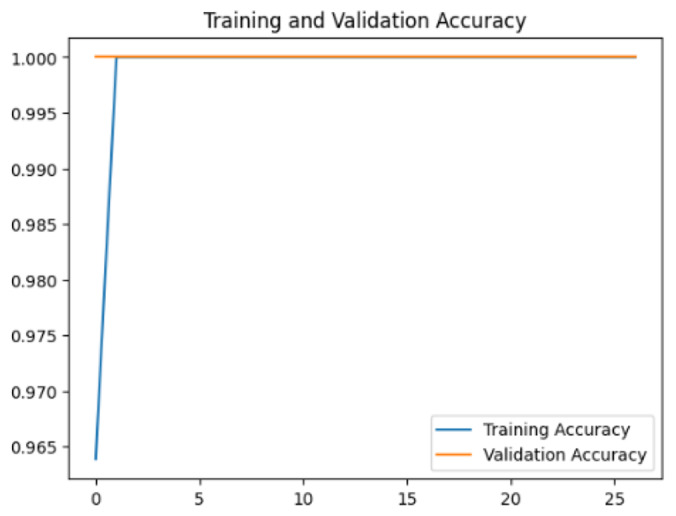	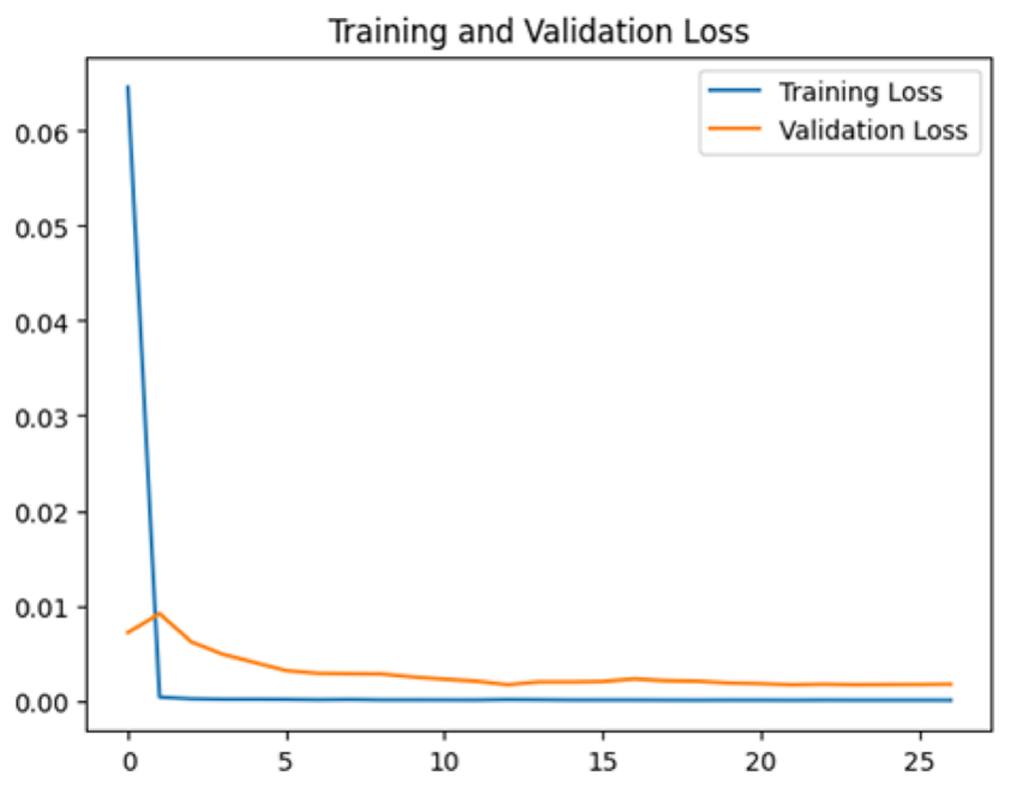

In addition, the confusion matrix for each model is shown in Table 4 to visualize how well the classification model works by showing the correct and incorrect predictions made by the model, in comparison with the actual answer. The confusion matrix in binary classification consists of four components *i.e.,* true positives (TP) is when the model correctly predicts the positive class; true negatives (TN) is when the model correctly predicts the negative class; false positives (Type-1 error) is when the model incorrectly predicts the positive class and false negative (Type-2 error) when the model incorrectly predicts the negative class (Saito & Rehmsmeier, 2015). InceptionV3 at LR 0.01, LR 0.001 and Xception at LR 0.0001 have made all correct predictions. Meanwhile, InceptionV3 and MobileNetV2 at LR 0.0001, Xception at LR 0.01 and LR 0.001, as well as VGG19 at all LR, have a Type-1 error in nest prediction. Whereas MobileNetV2 at LR 0.001 has a Type-2 error in nest prediction.

The confusion matrix in binary classification consists of four components: true positive (TP), when the model correctly predicts the positive class; true negative (TN), when the model correctly predicts the negative class; false positive (Type 1 error), when the model incorrectly predicts the positive class; and false negative (Type 2 error), when the model incorrectly predicts the negative class (Saito & Rehmsmeier, 2015). InceptionV3 at LR 0.01, LR 0.001 and Xception at LR 0.0001 all made correct predictions. InceptionV3 and MobileNetV2 at LR 0.0001, Xception at LR 0.01 and LR 0.001 and VGG19 at all LRs have a type 1 error in nest prediction. While MobileNetV2 at LR 0.001 has a type 2 error in nest prediction.

**Table 4:** Confusion matrix for the prediction models.

	Learning rate 0.01	Learning rate 0.001	Learning rate 0.0001
InceptionV3	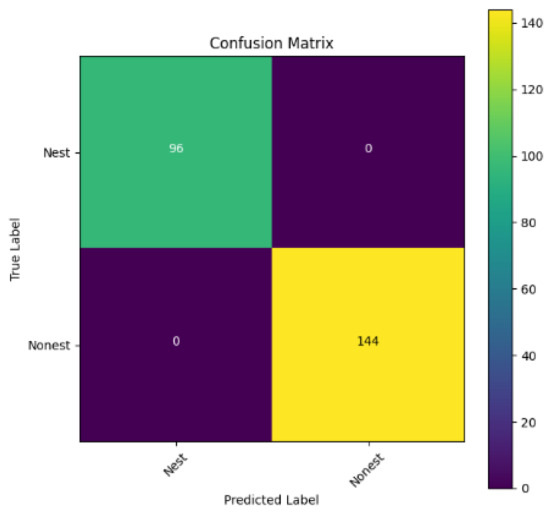	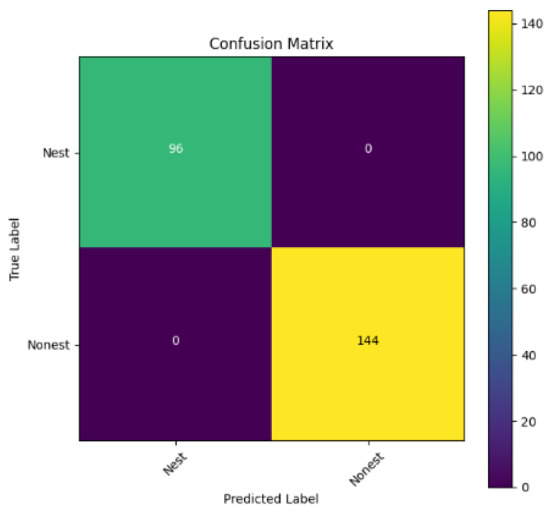	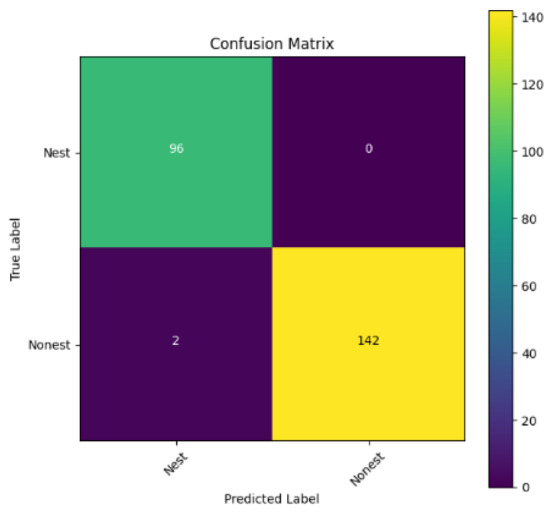
MobileNetV2	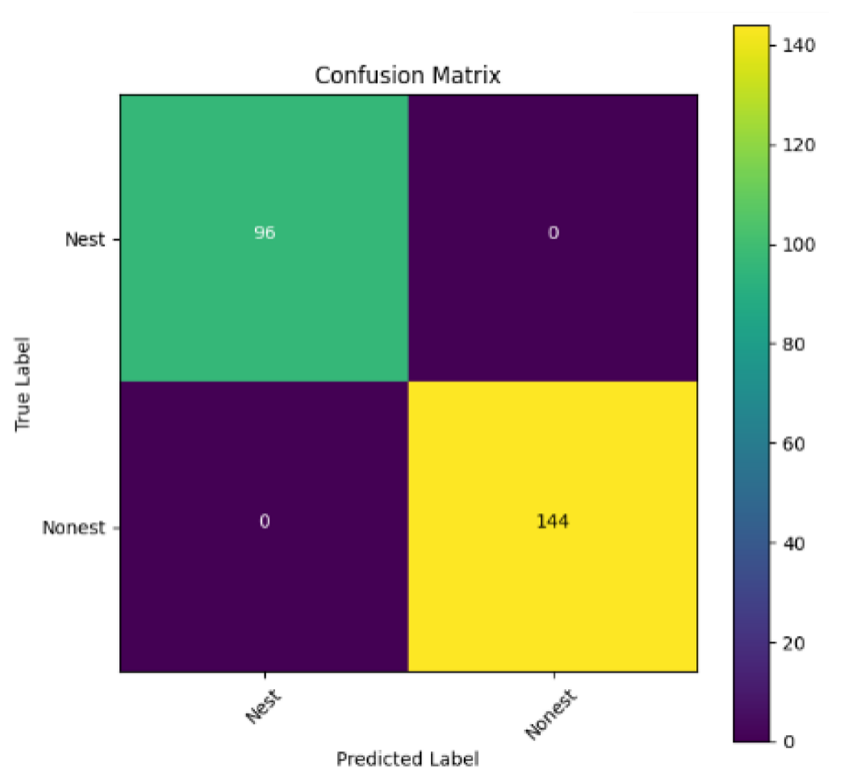	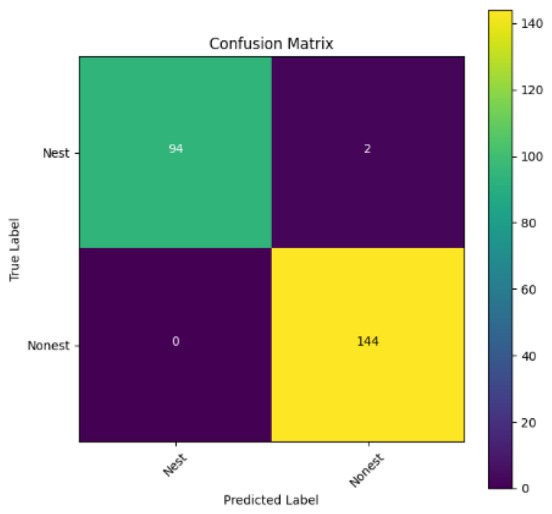	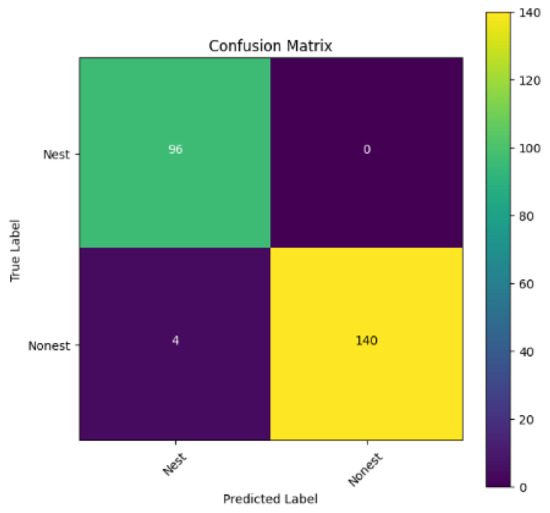
VGG19	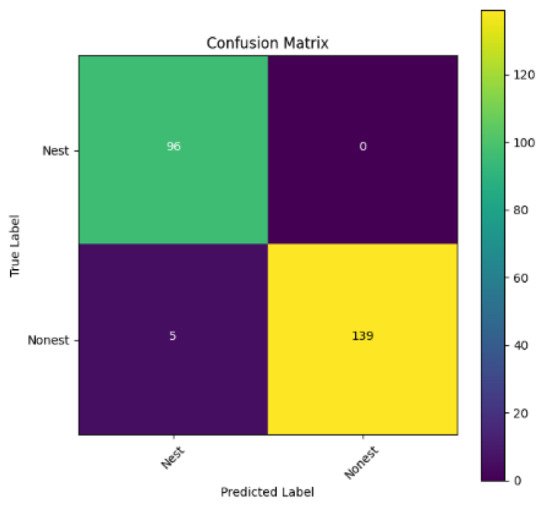	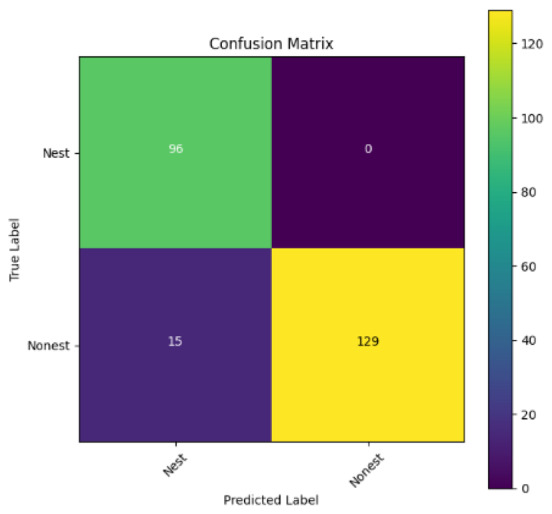	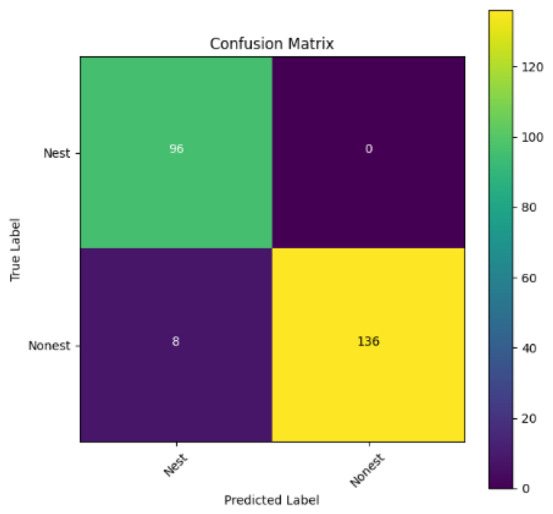
Xception	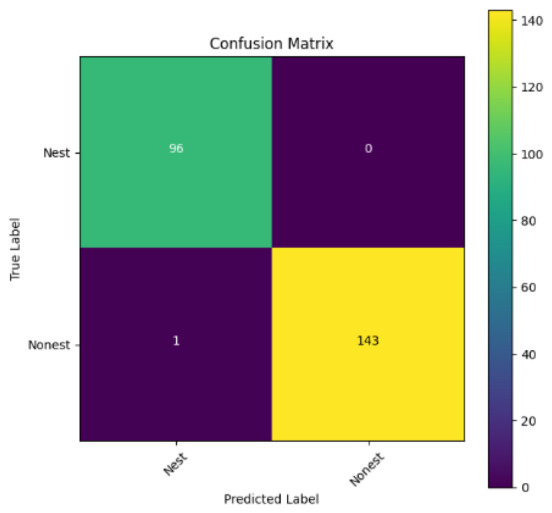	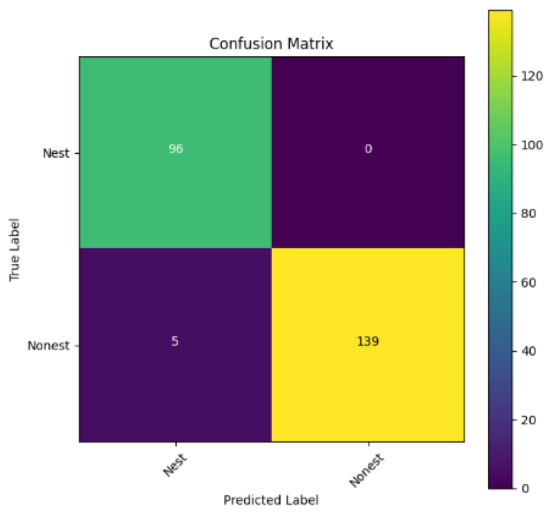	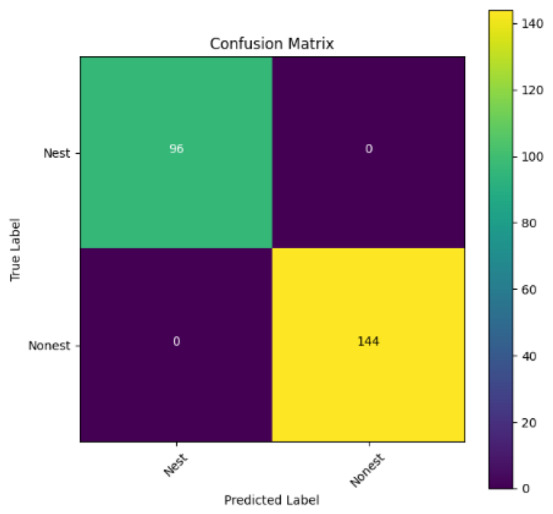

### Identification and visualization of input features

**Figure 5:**
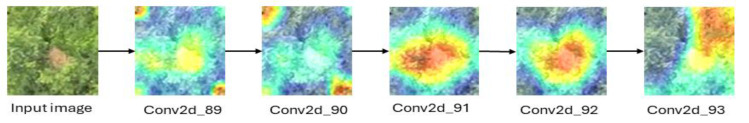
When processing the input aerial image of the orangutan nest, the edge of the nest (Conv2d_89 and 90) and the texture of the nest (Conv2d_91 to 93) are recognized step by step.

Heatmaps illustrate which parts of an image the model considers important by highlighting them in warm colors such as yellow, orange and red. Due to the superior overall performance of InceptionV3, five convolutional layers of the InceptionV3 architecture covering the low- and high-level features were used to visualize how the neural network identified the orangutan nest. Table 5 shows some examples of the convolutional layers of InceptionV3 compared to the original image of a human. The most common 2D convolutional layer “Conv2d” (Khan, 2019) is used to visualize the region used by the model for classification. The heatmaps derived from Conv2d_89 and Conv2d_90 highlighted the corners of the images and underlined subtle colors on the nest itself. In contrast, the nest was emphasized in the Conv2d_91 and Conv2d_92 heatmaps. In addition, the upper right corner of the image was emphasized in the heatmap derived from Conv2d_93. Based on the result, the neural network was able to identify the features of the nest –edge, shape and texture –reflected in the different intensities of warm color. As mentioned by LeCun, Bengio & Hinton (2015), there were blocks of low and high feature extraction in InceptionV3. Figure 5 shows an example of the original image used to extract the feature for classification.

**Table 5:** Heatmap of five convolutional layers from the InceptionV3 model to visualize the region used in image classification.

Conv2d_89	Conv2d_90	Conv2d_91	Conv2d_92	Conv2d_93
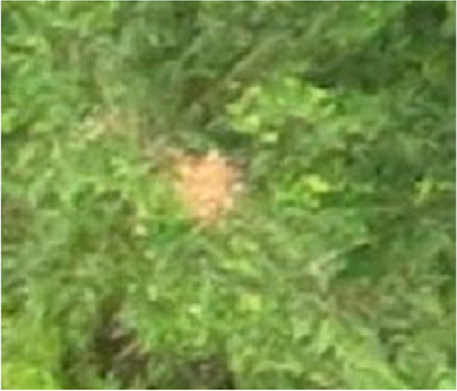	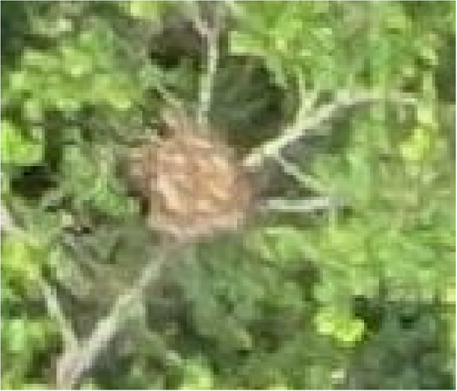	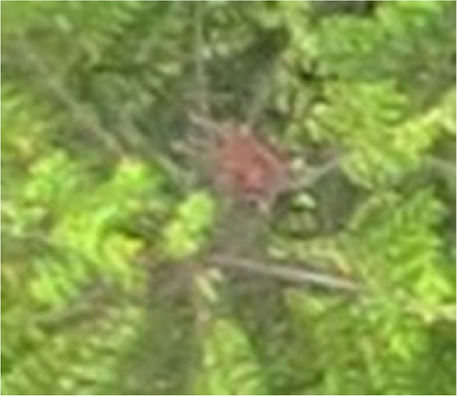	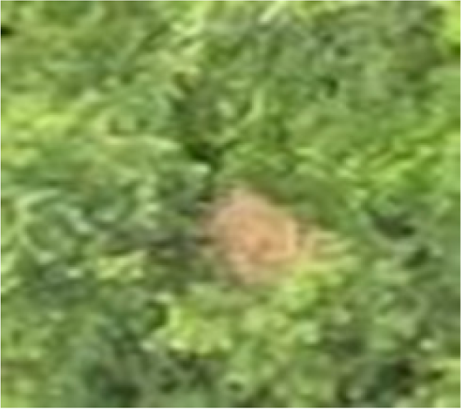	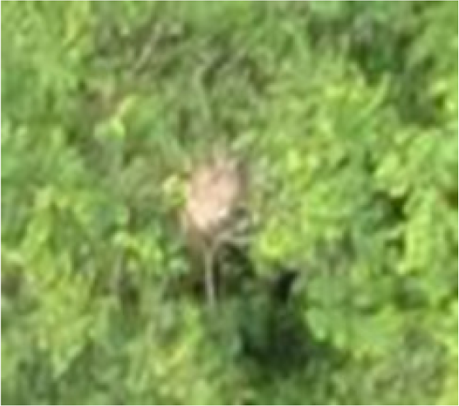
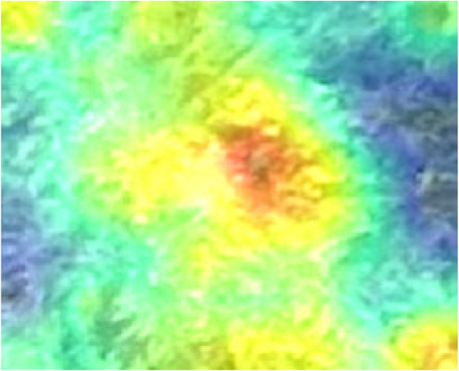	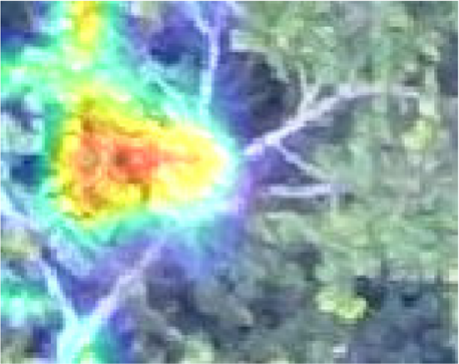	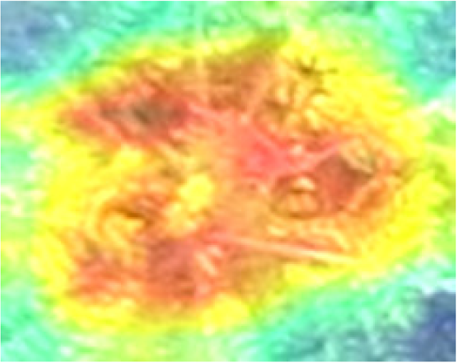	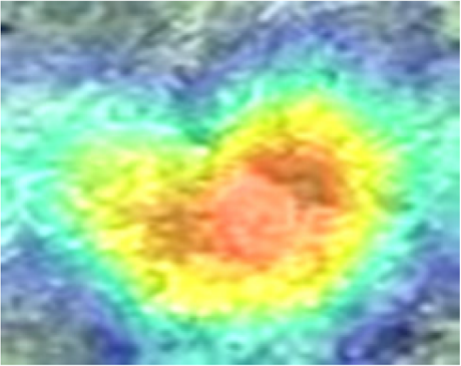	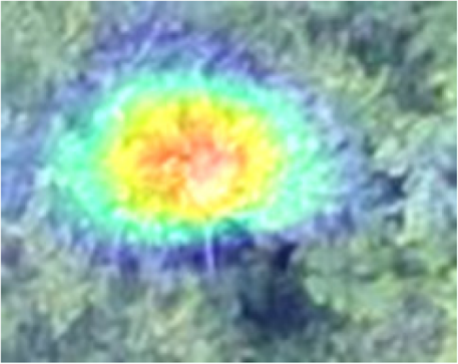

## Discussion

The increasing use of drones to monitor orangutan populations could be an excellent alternative to improve the monitoring and protection of orangutan populations (Burke et al., 2019). However, the enormous amount of data generated by UAV imagery, which needs to be identified and annotated by trained experts, poses a major time and labor-intensive challenge. Therefore, this study was conducted with the aim of evaluating the feasibility of using computer vision and DL to classify orangutan nests from UAV imagery.

This study focuses on image classification rather than object detection (Sharma, 2019). In particular, it supports the second stage of the two-stage object detection algorithm, which in this case involves the identification of the orangutan nest. The concept of two-stage detection consists of the first stage of detecting the object of interest (usually using the YOLO or SSD algorithm) and the second stage of a classifier by a DL algorithm (the DL models examined in this study). Although many data scientists or ML engineers have proposed only the YOLO algorithm, which can solve both localization (detecting the position of the object of interest on an image) and classification in one step, detecting and classifying an orangutan nest on aerial images of tree canopies is a great challenge in reality (due to the very similar patterns of tree canopies) and requires a large number of aerial images as training data. Therefore, the advantage of the DL model used in this study is that it is computationally more effective because the neural network focuses on classification tasks rather than two tasks in the object detection model. However, the output of the DL model was the result of labeling—images with nest and without nest—and the counting of the nest could only be done by another counting algorithm for the images placed in a folder with nest. The result of this orangutan nest recognition study is consistent with that of Chen et al. (2014), who integrated various AI methods, including ML, optimization algorithms and adaptive decision-making systems, to develop intelligent systems capable of performing complex orangutan nest detection tasks from UAV imagery. In addition, the current study on the use of DL architectures with feature extraction from the images has continued the study of Amran et al. (2023) who used hand-crafted feature extraction and multi-class classification with SVM for orangutan nest in Borneo. Although Teguh et al. (2024) attempted to use YOLOv5 and achieved a precision of 0.973 and a recall of 0.949 when recognizing the orangutan nest from the drone images, this study has shown that orangutan nest recognition can achieve higher accuracy and precision when using lower computational power (and focusing only on the classification task). In addition, this study has shown that unlike YOLO (single-stage recognition algorithm), the use of transfer learning (transferring weights and bias in the classification of ImageNet images to another classification task) also helps to overcome the problem of data scarcity associated with the lack of sufficient training examples. While counting nests from the ground is easier than locating and counting individual orangutans, drone surveys capture only a fraction of nests in aerial views. Nests under the canopy in dense forests are often missed, and fresh green nests or those in advanced decay stages are harder to detect in drone images (Andini et al. 2021). As a result, this may cause insufficient training data for model training. Despite their efficiency, UAV-based images often suffer from occlusion by dense tree canopies, making it difficult to detect nests that are clearly visible from the ground. In addition, variations in lighting conditions, camera angles and flight altitudes can lead to inconsistent image quality, which affects the accuracy of automatic detection models. In contrast, manual observation from the ground allows for more accurate inspection of nest features such as age, freshness and position, which are difficult to reliably assess from aerial imagery alone.

So far, this study is the first to compare four state-of-the-art pre-trained DL models—InceptionV3, MobileNetV2, VGG19 and Xception. The data was further augmented and the hyperparameters were refined by training for nest recognition from UAV imagery, resulting in high accuracies (>96%). The model performance result is in line with Ong & Hamid (2022); Ong et al. (2022), where InceptionV3 is the best model for this task, while VGG19 performs the worst. When comparing between the three learning rates, the learning rate (LR) of 0.001 achieved the optimal performance, with fewer problems related to overfitting and underfitting. InceptionV3 with LR 0.001 performed well and delivered all correct predictions.

It is worth noting that VGG19 performs the worst in this study, in contrast to other studies which showed that VGG19 performs better than InceptionV3 and MobileNet. A look at the layouts of VGG19 (Table 6) compared to InceptionV3 (Table 5) shows that VGG19 is not able to recognize the features of the orangutan nest, which could be the main reason for the poor performance. Nevertheless, there are previous studies that also show that VGG19 performs poorly. This emphasizes the need to compare DL models for a specific task.

**Table 6:** Heatmap of five convolutional layers from the VGG19 model to visualize the region used in image classification.

Block4_conv2	Block4_conv3	Block5_conv1	Block 5_conv2	Block5_conv3
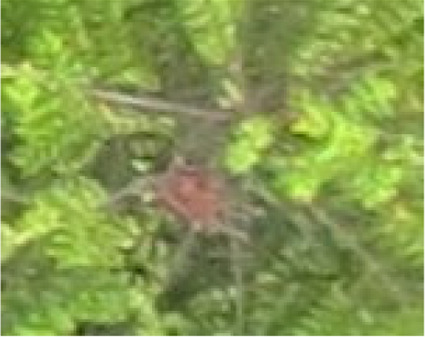	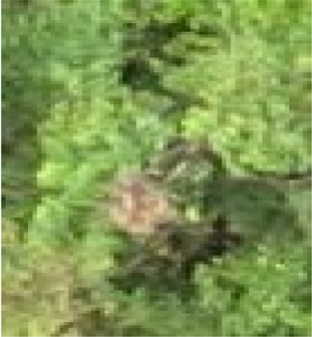	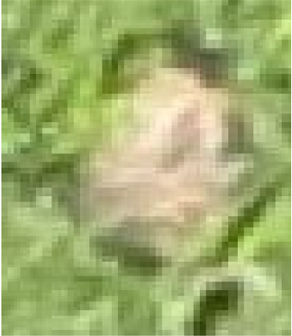	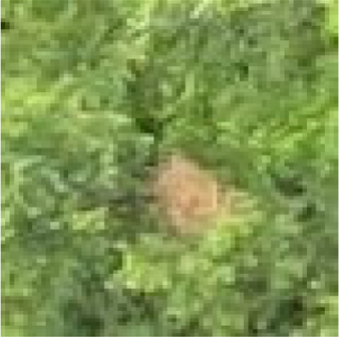	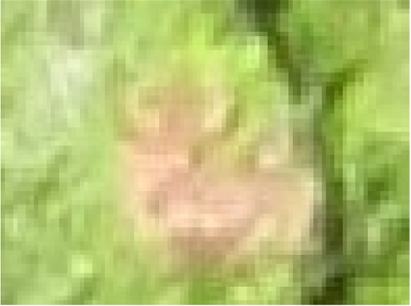
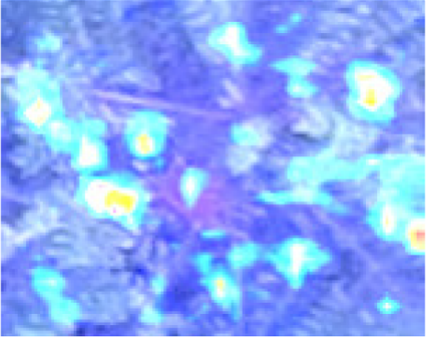	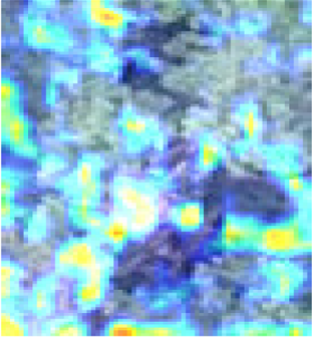	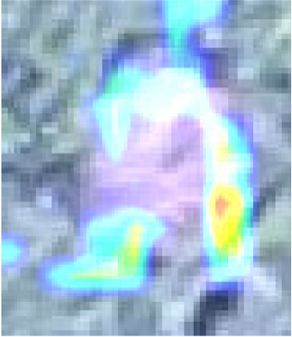	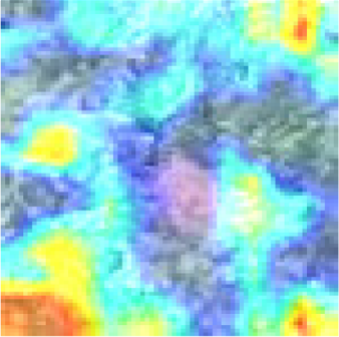	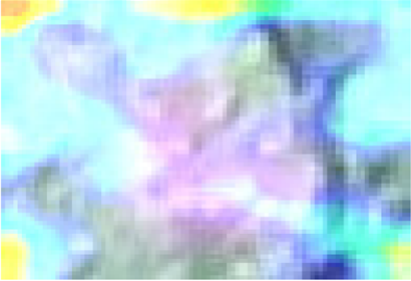

To interpret the result of the computer vision system for the orangutan nest, the layers of the architecture with Grad-CAM were visualized, which to our knowledge is also the first report. Using Grad-CAM, the region of biases and weights defined by the perceptron within the DL architecture was able to highlight the shape and texture of the orangutan nest, which was later used in the classification block for classification. Considering the similarity of the present study to the task of classifying the canopy of a forest, this study result was compatible with that of Nezami et al. (2020), who used a multilayer perceptron (MLP) to classify tree species using aerial images generated from RGB and hyperspectral (HS) images and achieved an accuracy of 99.6% with the best 3D CNN classifier. Moreover, the result of this study in classifying tree canopy with and without orangutan nests is consistent with that of Huang et al. (2023), who used ResNet, ConvNeXt, ViT and Swin Transformer and achieved at least 96% accuracy in classifying tree species from aerial images.

However, there are still many aspects that require further investigation and improvement. One of these is the quality of aerial images. As mentioned by Huang et al. (2023), the degradation of image quality and aerial images at different altitudes needs to be explored further. The key question for future study is to determine what altitude achieves the ideal balance between drone flight feasibility and image quality. In this study, for example, a fixed-wing drone with a Canon Power Shot S100 RGB CMOS sensor was used, which was flown at the highest point of the treetops at an altitude of 100 m. The image quality could be improved by using a multi-rotor UAV with better camera control. Image quality could also be improved by flying at a lower altitude where the camera is closer to the canopy and can capture more detail. However, this depends on the feasibility of the flight, where many factors determine the closest distance between the drone and the tree canopy, such as the availability of the crash sensor. With better image quality, further exploration can be conducted, such as classifying the nest decay stage of nests and increasing the ability to detect fresh green nests. Additionally, there is a need to augment both the quantity and diversity of aerial imagery to increase the robustness and subsequent generalization of the model. The diversity of the data could also include false positives and negatives in the training data to further improve the generalization of the model. Another important consideration is the deployment of the model to ensure its practical applicability. In the field for detecting and counting the number of orangutan nests. Additionally, building a model by local or regional dataset was always facing a challenge in generalizing good results for other similar datasets (*e.g.*, by using the DL model in this study to predict aerial images from Indonesia).

This study presents a comparative analysis of deep learning models for automatic detection of orangutan nests on aerial images. Although the results demonstrate the effectiveness of different deep learning models in classifying orangutan nests, we acknowledge several limitations that limit the novelty and broader applicability of our work. First, classification or object recognition using deep learning is already well established in computer science and machine learning techniques in various fields, including wildlife monitoring. Although our study provides a practical insight into the performance of the models, the comparison of existing models is inherently incremental rather than novel. Therefore, this work primarily contributes to the ability of the models to classify the orangutan “nests” from tree canopies, rather than quantitative data about the species’ ecology. Second, while analyzing the importance of features provides some insight into how different models “see” and interpret orangutan nests, further investigations—such as linking these results to field-based nest features or forest structure—were beyond the scope of this study. Future work could investigate how the interpretability of the models relates to ecological context or how it influences conservation decisions.

Many future studies will aim to improve the model, software and hardware. However, it is vital to ensure that these improvements consistently contribute to orangutan conservation. Streamlining orangutan survey and monitoring processes to be more cost and time efficient, alongside leveraging computer vision and DL models for automatic annotation of orangutan nests from aerial images, could significantly advance orangutan monitoring efforts.

## Conclusions

Our results show that InceptionV3 is able to classify the aerial images of orangutan nests with an accuracy and precision of 99%. The present study encourages further development of DL models for the automatic detection of orangutan nests from aerial UAV images. Further research and refinement in this area could lead to more time- and cost-efficient methods of identifying nests and thus monitoring the orangutan population. Nevertheless, additional data sets, especially from different forest types used by orangutans, such as forest patches within plantations, timber plantations, logged and unlogged forests, are crucial to improve the generalization of the model in the field. In the future, other remote sensing data such as through partnerships with other agencies could be incorporated to obtain more imagery and make significant improvements in this area.

### Ethics statement

The drone was deployed in the primary protection forest where no residents lived, and only images of the canopy were collected, so there was no risk to people’s privacy. The field study and the use of the drone for aerial photography were conducted in 2014 with permission from the Sabah Forestry Department under reference number (JPHTN/PP 100-22/4/KLT.11(44)).

## Additional Information and Declarations

### Field Study Permissions

The following information was supplied relating to field study approvals (*i.e.*, approving body and any reference numbers): The field study and the use of the drone to survey the orangutan nest were approved by the Sabah Forestry Department (reference number - JPHTN/PP 100-22/4/KLT.11(44)).

### Data Availability

The following information was supplied regarding data availability: The code is available at GitHub and Zenodo: – https://github.com/songguan26/Bornean-Orangutan-Nest-
- Song-Quan, O., & Donna, S. (2025). Bornean Orangutan Nest Identification Using Computer Vision and Deep Learning Models. In Bornean Orangutan Nest Identification Using Computer Vision and Deep Learning Models to Improve Conservation Strategies. Zenodo. https://doi.org/10.5281/zenodo.17452966. The dataset is available at figshare: Ong, Song-Quan (2025). Orangutan Nest and No-Nest. figshare. Figure. https://doi.org/10.6084/m9.figshare.29877266.v1.
